# Forward genetic screening identifies novel roles for N-terminal acetyltransferase C and histone deacetylase in *C. elegans* development

**DOI:** 10.1038/s41598-022-20361-x

**Published:** 2022-09-30

**Authors:** Rose Aria Malinow, Ming Zhu, Yishi Jin, Kyung Won Kim

**Affiliations:** 1 Department of Neurobiology, School of Biological Sciences, University of California, San Diego, La Jolla, CA 92093 USA; 2 Department of Life Science, Hallym University, Chuncheon, 24252 South Korea; 3 Multidisciplinary Genome Institute, Hallym University, Chuncheon, 24252 South Korea

**Keywords:** Caenorhabditis elegans, Development, High-throughput screening

## Abstract

Coordinating the balance between development and stress responses is critical for organismal survival. However, the cellular signaling controlling this mechanism is not well understood. In *Caenorhabditis elegans,* it has been hypothesized that a genetic network regulated by NIPI-3/Tibbles may control the balance between animal development and immune response. Using a *nipi-3(0)* lethality suppressor screen in *C. elegans*, we reveal a novel role for N-terminal acetyltransferase C complex *natc-1/2/3* and histone deacetylase *hda-4*, in the control of animal development. These signaling proteins act, at least in part, through a PMK-1 p38 MAP kinase pathway (TIR-1–NSY-1–SEK-1–PMK-1), which plays a critical role in the innate immunity against infection. Additionally, using a transcriptional reporter of SEK-1, a signaling molecule within this p38 MAP kinase system that acts directly downstream of C/EBP bZip transcription factor CEBP-1, we find unexpected positive control of *sek-1* transcription by SEK-1 along with several other p38 MAP kinase pathway components. Together, these data demonstrate a role for NIPI-3 regulators in animal development, operating, at least in part through a PMK-1 p38 MAPK pathway. Because the *C. elegans* p38 MAP kinase pathway is well known for its role in cellular stress responses, the novel biological components and mechanisms pertaining to development identified here may also contribute to the balance between stress response and development.

## Introduction

There is growing evidence of an intricate balance between promoting animal development and protection against stressful events. The trade-off between growth and stress response is conserved across many species^1–5^. The genetic networks involving this mechanism, however, are poorly understood.

In *C. elegans*, the convergence between mitogen-activated protein kinase (MAPK) regulators in development and innate immunity was recently discovered in studies of NIPI-3/Tribbles^6^. NIPI-3 was first identified in a screen for genes required for the induction of the innate immune response after fungal infection^7^. A partial loss of function of *nipi-3* causes animals to be susceptible to fungal and bacterial infection^7–11^. However, genetic null *nipi-3* mutants are larval arrest and lethal, showing that NIPI-3 is also required for animal development^6^. In these cases, NIPI-3 functions via regulation of p38 MAPK signaling (TIR-1–NSY-1–SEK-1–PMK-1 cassette) that regulates both development and immune response^6,7^. For example, a gain-of-function mutation in *nsy-1/*MAPKKK causes hyperinduction of PMK-1-dependent immune effectors and confers increased resistance to the bacterial pathogen *Pseudomonas aeruginosa*^12^. However, the *nsy-1* gain-of-function mutation also significantly delays animal development^12^. Here, we focus on the role of NIPI-3 in regulating animal development where loss of NIPI-3 causes increased expression of the transcription factor CEBP-1^6^. CEBP-1 then promotes the transcription of the MAPKK *sek-*1. Regulation of the expression and activity of this MAPKK is vital to the role of this pathway in innate immunity^13^, but is poorly understood in the context of development.

We have previously reported a forward genetic screen for suppressors of *nipi-3(0)* larval arrest and lethality, and identified CEBP-1, MAPK activated protein kinase (MAPKAPK) MAK-2, and the TIR-1–NSY-1–SEK-1–PMK-1 innate immune cassette to be negatively regulated by NIPI-3^6^. The identification of any additional regulators of this pathway is an important next step in understanding the NIPI-3-related genetic network. In this study, we identify such additional regulators by expanding the analyses of *nipi-3(0)* suppressor mutations. Here, we report the components of the N-acetyltransferase C (NatC) complex (NATC-1, NATC-2, and NATC-3) and the histone deacetylase (HDAC) HDA-4, to function with NIPI-3 to regulate animal development. Using a transcriptional reporter (*Psek-1::GFP*) as a readout of CEBP-1 activity, we gain insight into some of the interactions between identified regulators in this pathway. Our findings suggest a likely positive control of *sek-1* transcription by *pmk-1*/MAPK, *sek-1/*MAPKK, *mak-2*/MAPKAPK*.* Using a *nipi-3* suppressor screen and a *sek-1* transcriptional reporter, this study identifies novel regulators of development and identifies transcriptional regulation of a key MAPKK within a well-studied PMK-1 p38 MAPK pathway in its role in regulating animal development.

## Results and discussion

### Loss of N-terminal acetyltransferase C complex suppresses *nipi-3(0)* mediated larval arrest and lethality

The *nipi-3(0)* single mutant animals never reach adulthood, stop developing at the second or third larval stage, and eventually die between 5 and 10 days after hatch^6^. To identify genes that function with *nipi-3* to regulate animal development, we carried out two forward genetic screens to select for mutations that suppress the larval arrest and lethality phenotype of *nipi-3(0)* (Supplementary Fig. S1). In our previously reported visual-selection screen (Supplementary Fig. S1A), we isolated multiple mutations that affect 6 genes, including bZip protein CEBP-1, MAPKAPK MAK-2, and a highly conserved PMK-1 p38 MAPK pathway (TIR-1–NSY-1–SEK-1–PMK-1)^6^. In the screen relying on selection against PEEL-induced toxicity (Supplementary Fig. S1B), we expanded the mutagenized genome and isolated total 35 independent suppressor mutations, most of which affect CEBP-1 or MAK-2 (Supplementary Fig. S1C). This allowed for the identification of a novel functional domain within CEBP-1^14^. Interestingly, despite screening more animals and expanding the mutagenized genome, our screen with PEEL-induced selection found no new genes (Supplementary Figs. S1C–S1E). Here, we report two mutant alleles isolated from the visual-selection screen, *ju1369* and *ju1371.*

**Figure 1:**
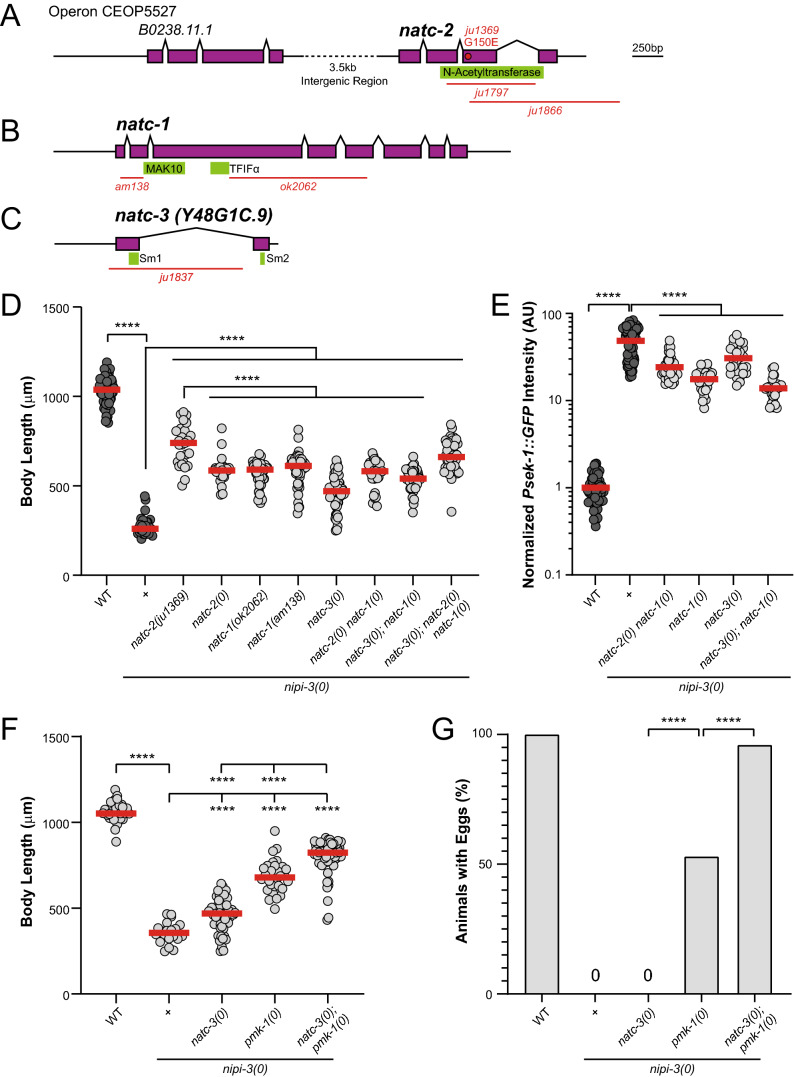
Loss of N-terminal acetyltransferase C complex suppresses *nipi-3(0)* larval arrest and lethality. (**A–C**) Illustrations of gene structure, missense allele, and deletion alleles. Exons are purple boxes, introns are angled black lines connecting exons, and the UTRs are black horizonal lines. Protein motifs are illustrated in green. Deletion alleles are indicated as red horizonal lines showing the region of DNA that is deleted. The missense mutation is indicated as a red dot in the exon where the mutation exists, allele name and amino acid substitution are labeled above the red dot. (**A**) Operon CEOP5527 containing *B0238.11.1* and *natc-2.* (**B**) *natc-1.* (**C**) *natc-3,* previously named *Y48G1C.9.* (**D**) Quantification of body length of animals 3 days post egg laid. Each dot represents a single animal, each red line represents the mean value. Some data are replicated from other Figures, shown in darker grey dots. Statistics: One-Way ANOVA with Tukey’s post hoc test. ****P < 0.0001. *nipi-3(0)* mutants have a short body length after 3 days of development post egg laid. This phenotype is rescued by *natc-2(ju1369), natc-2(ju1866), natc-1(ok2062), natc-1(am138)* or *natc-3(ju1837).* Compound knockout of *natc-1, natc-2* or *natc-3* (*natc-2(ju1866) natc-1(ok2062), natc-3(ju1837); natc-1(ok2062),* or *natc-3(ju1837); natc-2(ju1866) natc-1(ok2062))* does not rescue body length significantly more than each knockout on their own. (**E**) Quantification of *Psek-1::GFP* expression in animals 36 h post egg laid. Knockout of *nipi-3* substantially increases *Psek-1::GFP* expression, which is significantly reduced, but not brought to wild type (WT) expression levels by knockout of *natc-2(ju1797) natc-1(am138), natc-1(ok2062), natc-3(ju1837)* or *natc-3(ju1837); natc-1(ok2062).* Statistics: Kruskal–Wallis one-way ANOVA with Dunn's multiple comparisons test. **P < 0.01, ****P < 0.0001. (**F**) Quantification of body length of animals 3 days post egg laid. Double knockout of *natc-3* and *pmk-1* rescues body length significantly more than each knockout in *nipi-3(0)* background. Statistics: One-Way ANOVA with Tukey’s post hoc test. ****P < 0.0001. (**G**) Quantification of % of animals, measured for body length in (F), that contain eggs after 3 days of development post egg laying. Double knockout of *natc-3* and *pmk-1* rescues developmental delay defect of *nipi-3(0)* to almost WT levels. Statistics: Fisher’s exact test, two-tailed. ****P < 0.0001.

We first identified *ju1369* as a missense mutation in *natc-2,* which encodes a *C. elegans* ortholog of Naa30, the catalytic subunit of the NatC complex (Fig. 1A). Homology of Naa30 is defined by a N-acetyltransferase domain that is 76% similar and 62% identical between the *C. elegans natc-2* and human ortholog (Supplementary Fig. S2A). Protein N-terminal acetylation is one of the most common protein modifications, occurring on more than 80% of mammalian proteins^15^. This protein modification can regulate the subcellular localization, structure, and stability of the target protein^16–23^.

The *natc-2(ju1369)* mutation changes glycine 150 to glutamic acid in the N-acetyltransferase domain. Based on the crystal structure model of Naa30, this SNP (G150E in *C. elegans* NATC-2, G266E in hNaa30) is adjacent to the acetyl-CoA binding pocket of the protein (Supplementary Fig. S2B), suggesting that this mutation may affect the enzymatic activity of the protein. To determine if *natc-2(ju1369)* is a loss-of-function mutation, we generated an independent deletion allele (*ju1866)* in *natc-2* using CRISPR/Cas9 which deletes a large portion of the coding region of *natc-2* and is referred to as *natc-2(0)* hereafter (Fig. 1A). *natc-2(0); nipi-3(0)* animals are viable and fertile, indicating that *natc-2(0)* suppresses *nipi-3(0)* lethality and that *natc-2(ju1369)* is likely a loss-of-function mutation. Interestingly, the missense mutation *natc-2(ju1369)* rescues body length significantly better than *natc-2(0)*, indicating that *ju1369* has both loss-of-function and gain-of-function (neomorph) characteristics (Fig. 1D). This may be because in animals containing *ju1369*, the NatC complex forms and binds to its target proteins but is not functional. When NATC-2 is absent, the NatC complex may not form, however, other Nat complexes can compensate for the N-terminal acetylation by the NatC complex^24^.

The human NatC complex contains three subunits, which were identified by protein crosslinking and pulldown, first in yeast, then confirmed in human cells^25,26^. The three subunits of the mammalian NatC complex are the catalytic subunit Naa30, the ribosomal anchoring subunit Naa35, and the auxiliary subunit Naa38^25^. The *C. elegans* orthologues of Naa30 and Naa35 are NATC-2 and NATC-1, respectively. To determine if NATC-2 is acting through its function as a component of NatC complex, we tested if *natc-1(0)* has the same phenotype as *natc-2(0)*. We found that like *natc-2(0),* two independent deletion alleles of *natc-1, am138*^27^ and *ok2062*^28^ show suppression of *nipi-3(0)* lethality and developmental arrest phenotype (Fig. 1B and D). Furthermore, we created *natc-2(0) natc-1(0)* double mutants using CRISPR/Cas9 and found that this triple mutant *natc-2(0) natc-1(0); nipi-3(0)* does not show enhanced suppression over each of the double mutants (Fig. 1D), further suggesting that NATC-1 and NATC-2 function together in their role in animal development as a protein complex.

Based on sequence homology, we suspected that Y48G1C.9 encodes the auxiliary subunit (Naa38) of NatC (Supplementary Fig. S2C). We generated a deletion allele of Y48G1C.9 (*ju1837*) using CRISPR/Cas9 and made a double mutant with *nipi-3(0)*, which was viable and fertile (Fig. 1C and D). This suggests that Y48G1C.9 protein is likely functioning as a necessary component in a protein complex with NATC-1 and NATC-2. We therefore rename this gene *natc-3* based on sequence homology to Naa38 and phenotype in *nipi-3(0)* mediated development. As a result, we identified the gene encoding the third subunit of the *C. elegans* NatC, NATC-3, along with other NatC subunits.

### NatC mediates *nipi-3(0)*-induced larval arrest and lethality at least partially in parallel with the PMK-1 p38 MAPK pathway

To dissect the genetic interaction of NatC complex with the previously identified PMK-1 p38 MAPK pathway, we first asked if *natc-1*, *natc-2,* and *natc-3* function through the PMK-1 p38 MAPK pathway to regulate *nipi-3* developmental arrest using a *sek-1* transcriptional reporter (*Psek-1::GFP*). This transcriptional reporter is highly upregulated in *nipi-3(0)* animals, dependent on PMK-1 p38 MAPK pathway activity (Figs. 4 and 5A) and is described further below. In *natc-2(0) natc-1(0); nipi-3(0), natc-1(0); nipi-3(0), natc-3(0); nipi-3(0),* and *natc-3(0); natc-1(0); nipi-3(0)* animals, the expression level of *Psek-1::GFP* was significantly reduced in comparison to *nipi-3(0)*, but not nearly to the level seen in disruption of the PMK-1 p38 MAPK pathway (Figs. 1E and 5A; performed in a single experiment). This suggests that the NatC complex has a partial contribution to the regulation of *sek-1* transcription.

Furthermore, to test if NatC and PMK-1 function in parallel pathways, we produced *natc-3(0); pmk-1(0); nipi-3(0)* triple mutant animals and compared their body length and developmental timing to *natc-3(0); nipi-3(0)* or *pmk-1(0); nipi-3(0)* double mutants. The triple mutant animals showed better suppression of *nipi-3(0)* body length compared to either of double mutants (Fig. 1F). We also found that most (96%) *natc-3(0); pmk-1(0); nipi-3(0)* animals contained embryos after 3 days of development, whereas there was severe developmental delay in *natc-3(0); nipi-3(0)* animals (0% with embryos) and *pmk-1(0); nipi-3(0)* animals (53% with embryos) (Fig. 1G). As *natc-3(0)* and *pmk-1(0)* have an additive effect on suppression of *nipi-3(0)* induced developmental arrest, this suggests that *natc-3* and *pmk-*1 act at least partially in parallel. Taken together, the *C. elegans* NatC complex acts partially through the PMK-1 p38 MAPK pathway via regulation of *sek-1* transcription and at least partially in parallel to the PMK-1 p38 MAPK pathway in *nipi-3* dependent development.

### NIPI-3 has minimal effects on NatC expression

**Figure 2:**
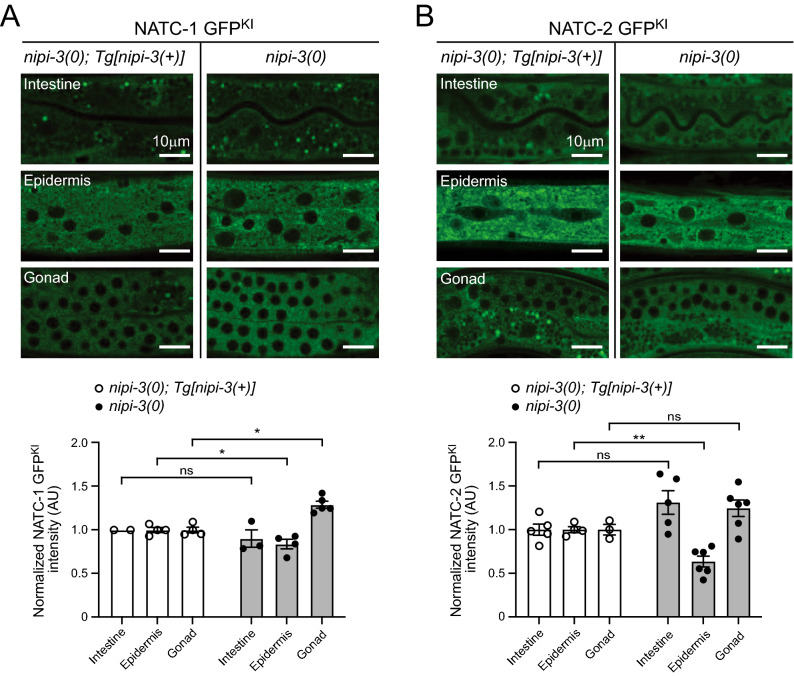
NIPI-3 has minimal effects on NATC-1 and NATC-2 expression. (**A, B**) Single plane confocal images of (**A**) NATC-1 GFP^KI^ (*ju1801*) and (**B**) NATC-2 GFP^KI^ (*ju1803*) in L3 animals. The left column of images is in the background of *nipi-3(0); Tg[nipi-3(*+*)]*, which has expression of WT *nipi-3* transgene (Tg), rescuing the larval arrest phenotype. Scale bar = 10 µm. The bar graph overlaid with a dot plot (bottom panels) represents quantification of the images with ImageJ software. Statistics: Mann–Whitney test. *ns*, not significant, *P < 0.05, **P < 0.01.

As NIPI-3 represses the expression of CEBP-1 and SEK-1 during development, we considered that NIPI-3 may also repress NatC expression. We generated N-terminal GFP knock-in alleles *ju1801* and *ju1803* for *natc-1* and *natc-2*, respectively (Supplementary Figs. S3A and S3B). Endogenous NATC-1 is broadly expressed in intestine, epidermis, and gonad, and localized in cytoplasm (Supplementary Figs. S4A–S4F; adult stages). Endogenous NATC-2 exhibits a similar expression pattern as NATC-1, but at varying levels in each tissue (Supplementary Figs. S4G–S4L). The endogenous expression pattern of NATC-1 is consistent with a transgene overexpressing GFP tagged *natc-1* genomic sequence^29^. To test if NIPI-3 regulates the expression of NATC-1 and NATC-2, double mutants of *nipi-3(0)* and NATC-1 GFP^KI^ *(ju1801)* or NATC-2 GFP^KI^ *(ju1803)* were produced. These animals remain arrested as larvae, indicating *ju1801* and *ju1803* do not disrupt the function of either gene. The absence of NIPI-3 was found to slightly alter the epidermal and gonadal expression of NATC-1 and epidermal expression of NATC-2, but the overall endogenous expression of NATC-1 and NATC-2 remained largely unaltered in the presence or absence of NIPI-3 (*nipi-3(0); [nipi-3(* +*)]* or *nipi-3(0)* background) (Fig. 2A and B; L3 stages), indicating that NIPI-3 has minimal effects on NATC-1 and NATC-2 expression.

In *C. elegans*, N-terminal acetylation mediated by homologues of NatA, NatB, and NatC plays a role in the regulation of animal development, stress tolerance, and entry into a stress resistant developmental stage^30^. About 74% of *C. elegans* proteins are N-terminal acetylated^24^, and null mutations to any members of NatA or NatB are lethal^31,32^, indicating that Nat complexes play an essential role in development. However, NatC seems to have a more nuanced role in regulation of development. Loss of function of NatC on its own does not seem to confer any overt developmental phenotype, although they do show significantly shorter lifespan than wild type^29^. *C. elegans* NatC was first identified in a screen for mutants with increased resistance to heavy metal toxicity^29^. Furthermore, loss of NatC was found to confer resistance to a broad spectrum of physiological stressors including oxidative stress and heat shock^29,30^. In this context, *nipi-3(0)* causes physiological stress by overexpressing CEBP-1 or hyperactivating the downstream p38 MAPK pathway, and resistance to this stress may be conferred by loss of NatC.

The exact mechanism by which NatC contributes to *nipi-3(0)* larval arrest is difficult to assess, at this point, given the many functions of NatC (e.g. regulating protein stability^19,20^; facilitating protein complex formation^22,23^; and controlling subcellular localization^16,21^). Additionally, the NatC complex can modify hundreds of individual proteins^33^, so it is likely that modification of many different proteins contributes to the *nipi-3(0)* phenotype. Our findings that NatC functions both through the PMK-1 p38 MAPK pathway as well as in an independent, parallel pathway support the view that N-terminal modification of many proteins likely contributes to the *nipi-3(0)* phenotype.

### Gain-of-function mutation in HDAC *hda-4* suppresses *nipi-3(0)* mediated lethality

**Figure 3:**
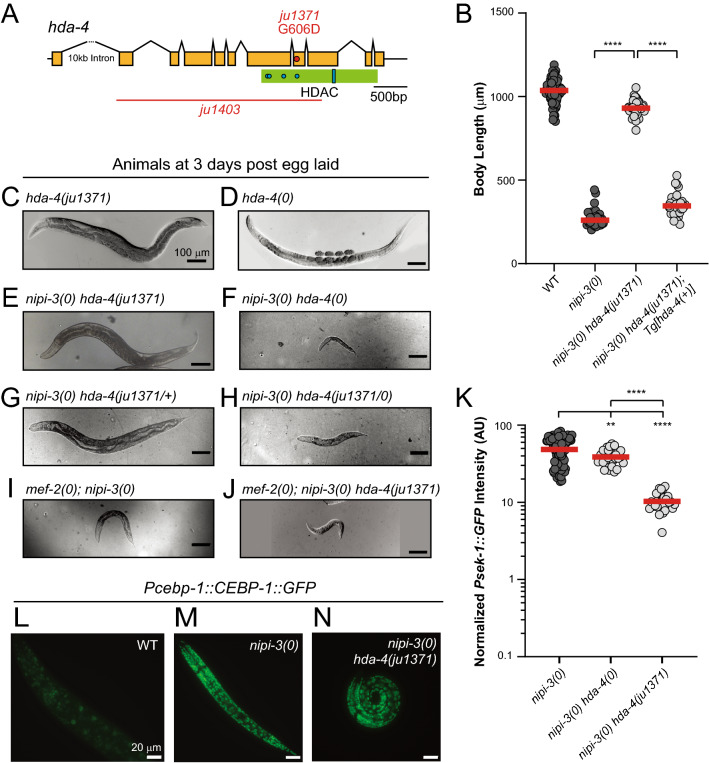
A gain of function mutation in histone deacetylase HDA-4 suppresses *nipi-3(0)* larval lethality and developmental arrest phenotype through PMK-1/p38 MAPK pathway. (**A**) Illustration of gene structure, missense allele, and deletion allele. There is a 10,113 bp first intron that is not illustrated to scale. Protein motif is illustrated in green. Blue dots illustrate zinc binding sites. The blue bar within the HDAC domain illustrates the LEGGY motif. Deletion allele is indicated as red horizonal line showing the region of DNA that is deleted. *ju1403* is a 3064 bp deletion. The missense mutation *ju1371* is indicated as a red dot in the exon where the mutation exists. (**B**) Quantification of body length of animals 3 days post egg laid. *nipi-3(0)* mutants have a short body length. This phenotype is rescued by *ju1371*. Overexpression of either HDA-4 (*oyIs73)* or CEBP-1 (*juIs418*) can reverse the phenotype of *hda-4(ju1371).* Statistics: One-Way ANOVA with Tukey’s post hoc test. *ns*, not significant, ****P < 0.0001. (**C–J**) Bright-field or DIC images of animals at 3 days post egg laid. Scale bar = 100 µm. (**C**) *hda-4(ju1371)* does not confer any gross developmental defects. (**D**) *hda-4(0)* animals are skinnier than WT but have no developmental delay. (**E**) Developmental arrest phenotype of *nipi-3(0)* is rescued by *hda-4(ju1371)*. (**F**) The developmental arrest phenotype of *nipi-3(0)* is not rescued by *hda-4(0)*. (**G**) Animal size, but not fertility is rescued by one copy of ju1371 and one copy of WT *hda-4*. (**H**) *ju1371* suppression is not dominant to *hda-4* null (**I**) *mef-2(0)* does not suppress *nipi-3(0)* (**J**) *mef-2* is required for *ju1371* suppression of *nipi-3(0)*. (**K**) Quantification of *Psek-1::GFP* expression in animals 36 h post egg laid. Knockout of *nipi-3* substantially increases *Psek-1::GFP* expression. Expression of *Psek-1::GFP* is significantly lower in rescued animals, *nipi-3(0) hda-4(ju1371),* in comparison to the animals that arrest during development, *nipi-3(0) hda-4(0).* Statistics: One-Way ANOVA with Tukey’s post hoc test, ***P < 0.001. (**L–N**) Fluorescent images of CEBP-1::GFP under the *cebp-1* promoter as a multicopy integrated reporter (*wgIs593*) in animals 24 h post hatch. Scale bar = 20 µm.

We mapped *ju1371,* isolated in the *nipi-3(0)* suppressor screen, to be a missense mutation in the catalytic domain of the HDAC *hda-4* (Fig. 3A)*.* Overexpression of wild-type HDA-4, with the *oyIs73* transgene^34^, reversed the suppression phenotype of *ju1371* (Fig. 3B, *nipi-3(0) hda-4(ju1371); Tg[hda-4(*+*)]* animals arrest during larval development), indicating that *hda-4(ju1371)* is the causal mutation of the *nipi-3(0)* suppressor phenotype. HDACs are enzymatic proteins that are most highly studied in their role in removing acetyl groups from histones, producing a closed chromatin state where transcription is less active^35^. HDACs also have non-enzymatic functions as transcriptional regulators via direct binding to transcription factors, corepressors, and protein-modifying enzymes^36,37^. Of the four classes of mammalian HDACs, class IIa HDACs are unique because they have extremely low deacetylase activity in biochemical assays due to a variation in a highly conserved motif in the zinc-dependent deacetylase catalytic domain^38^. In mammalian class IIa HDACs, the LEGGY catalytic motif, present in active deacetylase domains, is replaced by a LEGGH motif. The tyrosine of the LEGGY motif is necessary and sufficient to produce deacetylase activity^39^. Although the closest human homologues of *C. elegans* HDA-4 are class IIa HDAC7 (70% similar HDAC domain) and HDAC4 (65% similar HDAC domain), HDA-4 contains the active catalytic motif (LEGGY), whereas HDAC7/4 contain the inactive motif (LEGGH). This suggests that HDA-4 may be an active deacetylase, but biochemical studies must be performed with this *C. elegans* protein to experimentally determine its catalytic activity. *ju1371* changes a conserved glycine into an aspartic acid (G606D) within the HDAC domain of HDA-4 (Fig. 3A). This residue change is structurally adjacent to the catalytic LEGGY motif and just two amino acids away from the zinc binding site and within the HDAC domain, suggesting that this mutation may affect the enzymatic function of the protein.

To address whether *hda-4(ju1371)* is a gain or loss of function allele, we produced a deletion mutant *hda-4(ju1403)* by CRISPR/Cas9, which removes most of the coding region of *hda-4* and is referred to as *hda-4(0)* hereafter (Fig. 3A). We observed that both *hda-4(ju1371)* and *hda-4(0)* showed WT-like development, although *hda-4(0)* animals were noticeably thin (Fig. 3C and D). Interestingly, the *nipi-3(0) hda-4(0)* double mutant is larval lethal, resembling *nipi-3(0),* in stark contrast to the healthy *nipi-3(0) hda-4(ju1371)* double mutant (Fig. 3E and F), indicating that *hda-4(ju1371)* does not behave as a null allele. Furthermore, *hda-4(ju1371/*+*)* is able to partially suppress *nipi-3(0)* lethality as *nipi-3(0) hda-4(ju1371/*+*)* animals are able to reach adulthood but are sterile (Fig. 3G), indicating that *hda-4(ju1371)* dosage is relevant in rescue of larval arrest of *nipi-3(0*). On the other hand, one copy of *hda-4(ju1371)* is not able to suppress *nipi-3(0)* in the absence of *hda-4* as *hda-4(ju1371/0)* animals arrest in early larval stage (Fig. 3H), much like *hda-4(0)*. Thus, we conclude that *ju1371* is a semi-dominant gain-of-function allele and that *hda-4(ju1371)* dosage is relevant in rescue of larval arrest of *nipi-3(0)*.

### HDA-4 gain-of-function mutation acts through its role with MEF-2 and regulates PMK-1 p38 MAPK pathway activity

To further investigate the mechanism of *hda-4(ju1371)* suppression of *nipi-3(0)* larval arrest and lethality, we wanted to determine if this phenotype requires the transcription factor *mef-2*. In mammalian systems, HDACs and MEF2 form a protein complex that binds to DNA and represses transcription of MEF2 targets^40–42^. The HDAC and MEF2 interaction is conserved in *C. elegans*, where HDA-4 and MEF-2 form a complex that binds to DNA and inhibits transcription of targeted genes^34,43^. We found that *mef-2(0)* does not suppress *nipi-3(0)* larval arrest and lethality (Fig. 3I), but interestingly *mef-2(0); nipi-3(0) hda-4(ju1371)* mutants arrest in larval stage (Fig. 3J), indicating that *hda-4(ju1371)* requires *mef-2* to suppress *nipi-3(0)* lethality and developmental arrest phenotype*.* This suggests that *hda-4* may act through its role as a transcriptional repressor with *mef-2*.

We next asked if the suppression of *nipi-3(0)* by *hda-4(ju1371)* involves regulation of the p38 MAPK pathway. We found that the upregulation of *Psek-1::GFP* expression in *nipi-3(0)* background was dramatically reduced by *hda-4(ju1371),* but not by *hda-4(0)* (Fig. 3K). Thus, *hda-4(ju1371),* but not *hda-4(0),* reduces the transcription of *sek-1* in response to *nipi-3(0)*. We next asked if *hda-4(ju1371)* regulates *sek-1* transcription through the expression of the upstream transcription factor, CEBP-1. We found that the upregulation of CEBP-1 in response to *nipi-3(0)* is not blocked by *hda-4(ju1371)* (Figs. 3L–N), indicating that *hda-4(ju1371)* does not alter the expression of CEBP-1. These results suggest that *hda-4(ju1371)* functions through the PMK-1 p38 MAPK pathway by a mechanism independent of CEBP-1 expression level to regulate *nipi-3* dependent development.

The HDA-4–MEF-2 repressor complex has been previously described to regulate lifespan, chemoreceptor expression, lethargus (a sleep-like state), and transition to dauer stage in *C. elegans*^34,43^. We found that both *hda-4(ju1371)* and *mef-2* are required for suppression of *nipi-3(0)* developmental arrest. We also found that a dose-dependent rescue where one copy of wild-type *hda-4* and one copy of *hda-4(ju1371)* partially rescues *nipi-3(0)*. One possible explanation is that *ju1371* increases the repressive activity of the HDA-4/MEF-2 protein complex. This is likely not by modifying the HDA-4–MEF-2 interaction, since in mammalian systems, the binding between HDAC4 and MEF2 is independent of the histone deacetylase domain^41,44,45^. If *ju1371* increases repressive activity, identifying transcriptional targets of HDA-4 and MEF-2 through ChIP-seq analysis may provide insight into mechanisms underlying this phenotype. One model consistent with our data is that HDA-4–MEF-2 binds to the promoter of *sek-1* to repress its expression. However, we cannot rule out an indirect pathway regulating *sek-1* transcription.

A different model also consistent with our data is that HDA-4 normally has two independent functions: deacetylase actions on histones to regulate chromatin regulation as well as transcriptional regulation by binding with MEF-2. In this case, the *ju1371* mutation may inhibit the deacetylase activity, but leave the function of the HDA-4–MEF-2 protein complex intact. Attempts to test the possibility using an engineered HDA-4 (LEGGY to LEGGH) mutant have not yet been successful. Thus, the mechanism by which *ju1371* changes the function of HDA-4 remains unclear.

Here, we report that HDA-4, the homolog of human HDAC4/7 (class IIa HDAC), genetically interacts with *mef-2* and *nipi-3* to regulate development in *C. elegans* through a highly conserved PMK-1 p38 MAPK pathway. This provides a novel link between HDA-4–MEF-2 gene regulation and the PMK-1 p38 MAPK pathway. It is notable that many previous genetic screens for regulators of the PMK-1 p38 MAPK pathway have not identified this genetic interaction. Thus, subtle changes in forward genetic screens may find other regulators of this or other highly studied pathways.

### Transcriptional reporter of *sek-1,* as a functional readout of CEBP-1 activity, reveals novel regulations within p38 MAPK pathway

**Figure 4:**
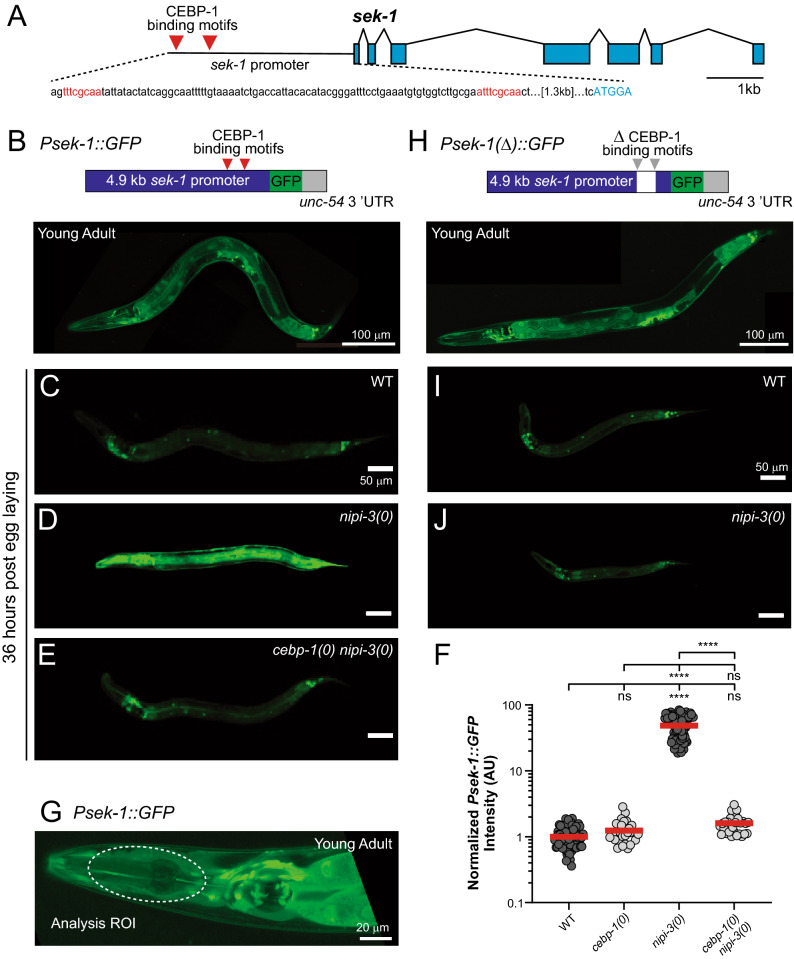
Transcriptional reporter of *sek-1* is a functional readout of CEBP-1 activity in the NIPI-3 dependent signaling network. (**A**) Illustration of the promoter and coding region of *sek-1.* The promoter region includes two identified CEBP-1 binding motifs (red triangles in illustration and red letters in sequence). Exons are pale blue boxes, introns are angled black lines connecting exons, and the 5’UTR is the black horizontal line. (**B**) Single plane confocal image of GFP expression driven by 4870 bp upstream sequence of *sek-1* as a multi-copy integrated reporter (*juIs559)* in L4 stage animal. Scale bar = 100 µm. (**C–E**) Single plane confocal images of *Psek-1::GFP* in (**C**) WT, (**D**) *nipi-3(0)* and (**E**) *cebp-1(0) nipi-3(0)* backgrounds (36 h post egg laid). Scale bars = 50 µm. (**F**) Quantification of *Psek-1::GFP* expression in animals 36 h post egg laid. Statistics: One-Way ANOVA with Tukey’s post hoc test. *ns,* not significant, ****P < 0.0001. (**G**) Single plane confocal image of *juIs559* in the head of a L4 stage animal. ROI used for quantification is marked with white dashed circle (see Methods for details). Scale bar = 20 µm. (**H**) Single plane confocal image of GFP expression driven by 4870 bp upstream sequence of *sek-1,* with two identified CEBP-1 binding motifs deleted, called *Psek-1(Δ)::GFP*, as a multi-copy extrachromosomal array (*juEx7617)* in L4 stage animal. Expression pattern is not altered by the deletion of the two CEBP-1 binding sites. Scale bar = 100 µm. **(I, J)** Single plane confocal images of *Psek-1(Δ)::GFP* in (**I**) WT and (**J**) *nipi-3(0)* backgrounds (36 h post egg laid). Scale bars = 50 µm*.*

Our previous study performed CEBP-1 ChIP-seq analyses to identify putative transcriptional targets and reported that the promoter of *sek-1,* encoding a MAP2K, contains two CEBP-1 binding sites 1.3 kb upstream from the start codon (Fig. 4A)^6^. The regulation of *sek-1* transcription by CEBP-1 was also validated with quantitative RT-PCR analysis of *sek-1* mRNA transcripts in wild type, *nipi-3(0)* and *cebp-1(0) nipi-3(0)* animals^6^. Our previous genetic screen identified a highly conserved PMK-1 p38 MAPK pathway as essential for developmental arrest caused by *nipi-3(0)*^6^*.* To characterize signaling linking NIPI-3 to this PMK-1 p38 MAPK pathway, we developed a transgenic *Psek-1::GFP* reporter (*juIs559*) using 4.9 kb upstream sequence of *sek-1* to drive expression of GFP (Fig. 4B). In wild-type animals expressing *Psek-1::GFP*, we observed a pattern of low levels of diffuse GFP fluorescence in all developmental stages and in multiple tissues including nervous system, intestine, and uterine muscles, but not body wall muscles or pharyngeal muscles (Fig. 4B). Within the nervous system, GFP was expressed in many neurons in ganglia in the head and tail, and touch receptor neurons. In *nipi-3(0)* larvae 36 h after egg laying, GFP expression was much greater throughout the animal in comparison to wild-type animals (Fig. 4C–G). As expected, *cebp-1(0) nipi-3(0)* animals have wild-type level expression of *Psek-1::GFP* (Fig. 4E). This regulation is due to the direct binding of CEBP-1 to the promoter of *sek-1* because another reporter, *juEx7617[Psek-1(Δ)::GFP*], in which the CEBP-1 binding sites were deleted from *Psek-1::GFP,* displayed no increase in the expression in *nipi-3(0)* animals and the deletion did not change the expression level and pattern in a wild-type background (Fig. 4H–J)*.* Combined with the ChIP-seq analyses, these results show that upregulation of *Psek-1::GFP* in *nipi-3(0)* is mediated by CEBP-1 binding to the *sek-1* promoter. Thus, CEBP-1 activity levels in this NIPI-3 regulated pathway can be measured, at least in part, with the *sek-1* transcriptional reporter, which we used to examine the complexity of the signaling downstream of NIPI-3 and CEBP-1.

**Figure 5:**
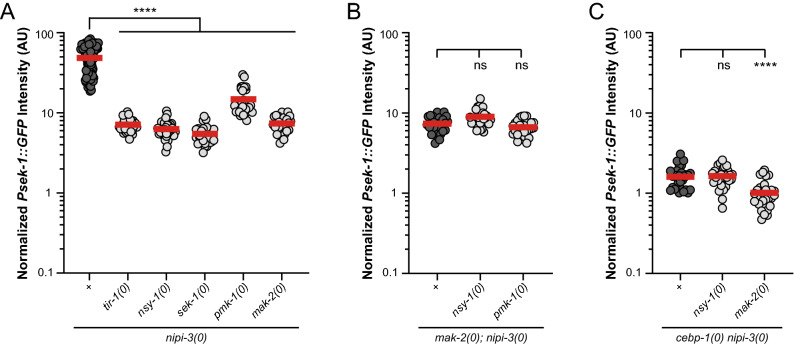
Transcriptional reporter of *sek-1* suggests *pmk-1*/MAPK and *mak-2*/MAPKAPK feedback regulation of transcription of *sek-1*. (A-C) Quantification of *Psek-1::GFP* expression in animals 36 h post egg laid. Null alleles in this figure are *nipi-3(ju1293)*, *cebp-1(tm2807), tir-1(qd4), nsy-1(ok593), sek-1(km4), pmk-1(km25),* and *mak-2(ok2394)*. Statistics Kruskal–Wallis one-way ANOVA with Dunn's multiple comparisons test. *ns*, not significant, **P < 0.01, ****P < 0.0001. (**A**) Knockout of *nipi-3* substantially increases *Psek-1::GFP* expression, which is significantly reduced by null mutations to any member of this MAPK pathway*. pmk-1(0)* is significantly weaker at suppressing *Psek-1::GFP* expression in comparison to all other knockouts. *Psek-1::GFP* expression in *mak-2(0); nipi-3(0)* is not significantly different than *nsy-1(0), tir-1(0),* or *sek-1(0)* with *nipi-3(0),* indicating that it is likely not downstream of *pmk-1* in this pathway regulating *sek-1* expression. (**B**) Compound mutants of *mak-2(0); nipi-3(0)* with *nsy-1(0)* or *pmk-1(0)* do not confer greater *Psek-1::GFP* suppression than each of the single mutants. (**C**) Compound mutants of *cebp-1(0) nipi-3(0)* with *nsy-1(0)* or *mak-2(0)* do not confer greater *Psek-1::GFP* suppression than each of the single mutants.

TIR-1 and NSY-1 increase SEK-1 activity^46–48^ and we found that *tir-1(0)* or *nsy-1(0)* significantly reduces the transcriptional upregulation of *sek-1* in *nipi-3(0)* (Fig. 5A). This suggests that decreasing SEK-1 activity decreases the transcription of *sek-1*. A positive regulation of *sek-1* transcription by SEK-1 is further supported by our finding that *sek-1(0)* significantly reduces *Psek-1::GFP* expression in *nipi-3(0)* (Fig. 5A). Thus, *sek-1, tir-1* and *nsy-1* contribute to the upregulation of *sek-1* transcription in the context of *nipi-3(0)*. *pmk-1(0)* also reduces the high levels of *Psek-1::GFP* observed in *nipi-3(0)* although not as much as *sek-1(0)* (Fig. 5A), indicating that *pmk-1* also positively regulates *sek-1* transcription. This feedback regulation was unexpected because in the context of innate immunity, many studies have placed SEK-1 function upstream of PMK-1^7,8,11,12,49–52^. Our previous study also showed that *sek-1* is required for PMK-1 phosphorylation in response to *nipi-3(0)*, placing *pmk-1* downstream of *sek-1* in this pathway during development^6^*.* Our observation that *pmk-1(0)* has a significantly weaker effect on *sek-1* transcription than *sek-1(0)* (Supplementary Fig. S5) suggests that SEK-1 regulates its own transcription, at least in part, independently from *pmk-1*.

We also found that removing *mak-2* produces similar *Psek-1::GFP* levels as removing *tir-1, nsy-1*, or *sek-1* (Fig. 5A), suggesting that *mak-2* also provides positive feedback to *sek-1* transcription. Notably, removing *mak-2* has stronger effects than removal of *pmk-1*. This was surprising because in mammalian systems, MAPKAPK (*i.e.,* MAK-2) are downstream of p38 MAPK kinases (*i.e.,* PMK-1)^53,54^, so we would expect *mak-2(0)* to phenocopy *pmk-1(0)*. In *C. elegans, mak-2* functions downstream of *pmk-3*/p38 MAPK in axon regeneration, synapse formation, muscle extension, cell fate patterning, and lifespan extension after mitochondrial disruption^55–58^. Our results suggest that MAK-2 activity in this pathway is regulated, at least in part, through signaling that does not depend on *pmk-1*. These findings suggest that the MAPK signaling participating in *C. elegans* development is not the linear pathway previously described, but rather a network containing numerous unexpected positive feedback loops with SEK-1 playing a central role.

### *mak-2*/MAPKAPK promotes transcription of *sek-1*/MAPKK, independently of *cebp-1* and is not downstream of NSY-1–SEK-1–PMK-1 signaling

To determine the genetic interaction of *mak-2* in the PMK-1 p38 MAPK pathway in *nipi-3* dependent development, we asked whether *mak-2* acts in parallel with *nsy-1* or *pmk-1.* To this end, we quantified the expression of *Psek-1::GFP* in wild type, *nsy-1(0)* or *pmk-1(0)* in the background of *mak-2(0); nipi-3(0)* and found that neither *nsy-1(0)* nor *pmk-1(0)* causes further suppression of *sek-1* transcription (Fig. 5B), suggesting that these genes are not functioning in parallel to regulate *sek-1* transcription in this pathway.

We then asked whether *mak-2* or *nsy-1* act in parallel with *cebp-1,* by producing triple mutants of *mak-2(0); cebp-1(0) nipi-3(0)* and *nsy-1(0); cebp-1(0) nipi-3(0)* and quantifying *Psek-1::GFP*. Of note, removing *cebp-1* almost completely blocks the effect of *nipi-3(0)* on *Psek-1::GFP* expression. We found that removing *nsy-1* shows no additive effect on the *sek-1* transcription in *cebp-1(0) nipi-3(0)* background, while removing *mak-2* shows a further suppression (Fig. 5C). This suggests that *nsy-1* does not have effects on *sek-1* transcription independent of *cebp-1*, while *mak-2* promotes *sek-1* transcription independent of *cebp-1*.

**Figure 6:**
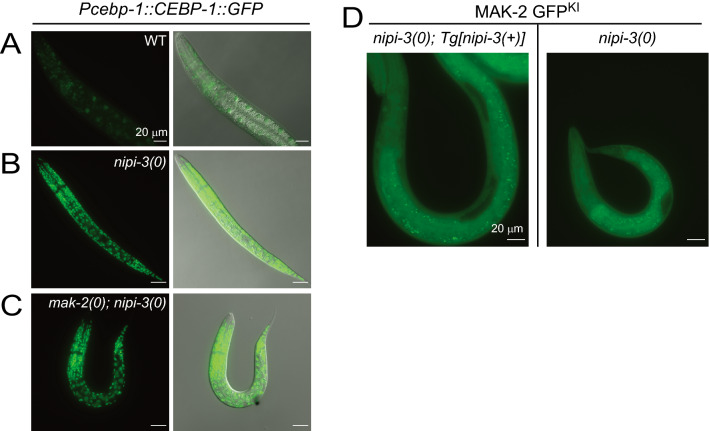
*mak-2* is not required for upregulation of CEBP-1 in response to *nipi-3(0)* and MAK-2 localization is unaltered by *nipi-3(0).* (**A**–**C**) Fluorescent and DIC images of CEBP-1::GFP under the *cebp-1* promoter as a multicopy integrated reporter (*wgIs593*) in animals 24 h post hatch. Scale bar = 20 µm. CEBP-1::GFP is expressed at low levels in WT background, while highly upregulated in *nipi-3(ju1293)* or in *mak-2(tm2927); nipi-3(ju1293)*. (**D**) Fluorescence images of MAK-2 GFP^KI^ (*ju1851)* in animals 24 h post hatch. The left column of image is in the background of *nipi-3(0); Tg[nipi-3(*+*)]* and the right column of image is in the background of *nipi-3(0).* GFP is expressed throughout the animal but is noticeably absent from the gonad. Scale bars = 20 µm.

We next tested if *mak-2* regulates the expression of CEBP-1 in the context of *nipi-3(0).* MAK-2 has been reported to act upstream of CEBP-1*,* increasing CEBP-1 expression by stabilizing its mRNA^55^. Using a strain expressing the translational reporter of *cebp-1* (*wgIs563[Pcebp-1::CEBP-1::GFP])*^59^, we tested the effect of loss of *mak-2* on CEBP-1 expression. As previously described, the expression of CEBP-1::GFP is seen within nuclei throughout the body in all tissues (Fig. 6A)^59^. *nipi-3(0)* animals have extremely high levels of the CEBP-1 reporter in all tissues (Fig. 6B) and *mak-2(0); nipi-3(0)* animals remain high as well (Fig. 6C). Thus, *mak-2* is not required for the upregulation of CEBP-1 in *nipi-3(0)* animals.

Next, we examined if MAK-2 activity is regulated by *cebp-1* in the context of *nipi-3(0)*. To test this, we took advantage of the fact that the localization of MAPKAPK (*i.e.,* MAK-2) is controlled by their phosphorylation state. In mammalian systems, when phosphorylated and active, the protein localizes to the cytoplasm and when unphosphorylated and inactive, it is sequestered in the nucleus^60^. Indeed, such regulation of localization has been shown in *C. elegans* neurons using transgenic constructs overexpressing phospho-mimetic or phospho-dead variants of MAK-2 driven by a neuronal specific promoter^55^. To visualize MAK-2 localization, we produced a strain, MAK-2 GFP^KI^ *(ju1851)*, in which the endogenous MAK-2 protein was tagged with GFP (Supplementary Fig. S3C). We observed expression of MAK-2 GFP^KI^ in both the cytoplasm and nucleus of multiple tissues including epidermis, muscle, intestine, and neuronal cells. We compared the expression of MAK-2 GFP^KI^ in *nipi-3(0); Ex[nipi-3(*+*)]* and *nipi-3(0)* mutant where CEBP-1 expression is low and high, respectively. We found no obvious changes in MAK-2 GFP^KI^ abundance or subcellular localization (Fig. 6D), suggesting that MAK-2 activity is not downstream of CEBP-1. If *mak-2* were downstream of *sek-1* via *pmk-1* as previous models of MAPK signaling would suggest, upregulating CEBP-1 should have produced a strong increase of MAK-2 activity. This raises the possibility that MAK-2 is not a downstream output of NSY-1–SEK-1–PMK-1 signaling, but rather regulates the activity of this pathway independently of CEBP-1.

**Figure 7:**
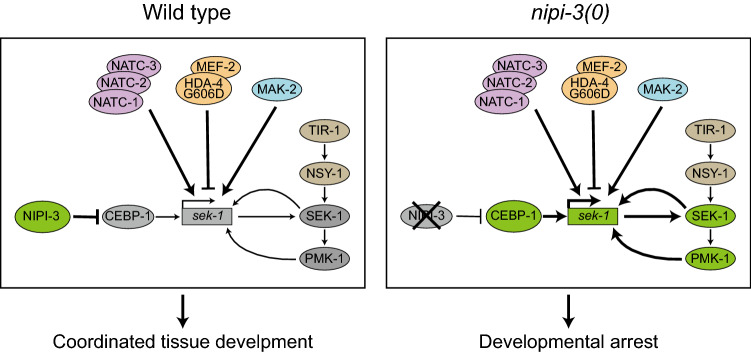
Hypothesis of signaling downstream of NIPI-3/Tribbles. A number of regulators downstream of NIPI-3 influence *sek-1* transcriptional regulation. The NATC complex *natc-1/2/3* and HDAC *hda-4* are identified as novel components in the control of animal development in this study. Furthermore, positive feedback mechanisms of *mak-2, pmk-1,* and *sek-1* forms a complex pathway to regulate proper animal development. The findings on the *nipi-3(0)* background led us to the wild-type scenario. See main text for details.

A transcriptional reporter of *sek-1* allowed us to identify novel feedback regulation from *pmk-1* and *mak-2*. Although these genes (*tir-1, nsy-1, sek-1, pmk-1*) have been studied extensively in their role in innate immunity, to our knowledge, no previous studies have identified feedback regulation from *pmk-1* or *mak-2* to *sek-1* transcription (Fig. 7 for models). Similarly, Wu and colleagues were able to identify feedback by interrogating expression of members within the PMK-1 signaling pathway in response to intestinal infection^11^. They found that in the context of infection, like during animal development, CEBP-1 transcription is negatively regulated by NIPI-3. Interestingly, they found that the transcriptional targets of CEBP-1 in response to infection differ from its transcriptional targets during development^11^. This agrees with previous observations that the transcriptional targets of CEBP-1 differ based on the circumstances^55,61^.

## Conclusions

For an organism to survive, the balance between growth and stress responses must be managed. An increasing body of evidence supports the idea that convergent networks control animal immunity and growth. In a prior study, we proposed that the NIPI-3–CEBP-1 axis is a critical mechanism for controlling immune effector expression in *C. elegans* during development^6^*.* In this study, we identified novel roles of the NatC complex *natc-1/2/3* and HDAC *hda-4* in regulation of animal development downstream of NIPI-3/Tribbles. Many forward genetic screens have been performed to identify genes required for innate immune response in *C. elegans* using bacterial, viral, and fungal infections as well as xenobiotics^7,8,12,62–68^, but neither NatC nor HDAC have been identified as regulators of the PMK-1 p38 MAPK pathway. Thus, using this unique sensitized background, we were able to identify previously unknown regulation of a highly studied p38 MAPK pathway in the context on development. The role of these newly identified regulators in innate immunity will require more research in the future.

*nipi-3(0)* produces extremely high levels of the transcription factor CEBP-1, which binds a conserved DNA motif in many genes regulating development and stress response in *C. elegans*^6^. The overexpression of CEBP-1 leads to developmental arrest, likely as an effect of the upregulation of genes downstream of CEBP-1 such as *sek-1.* Since *sek-1(0)* prevents the larval arrest of *nipi-3(0)* animals, *sek-1* expression is likely essential to the developmental arrest of the animals. Therefore, we produced a transcriptional reporter of *sek-1,* which allowed us to identify novel feedback regulation from *pmk-1* and *mak-2* as well as *sek-1* itself in the context of development. We have found regulatory feedback loops within this PMK-1 p38 MAPK pathway to be so interconnected that it will be challenging to design experiments to further dissect this pathway. Indeed, it is inherently challenging to resolve feedback loops within signaling pathway^69^.

## Methods

All experimental protocols were approved by the University of California, San Diego and all methods were carried out in accordance with relevant guidelines and regulations. All experiments were conducted in compliance with the ARRIVE guidelines.

### *Caenorhabditis elegans* strains and genetics

Strains were maintained on NGM plates at 20 °C as described previously^70^. Genetic crosses were performed using standard methods and genotypes for all mutations were confirmed using PCR or Sanger sequencing. Genotypes of strains are summarized in Supplementary Table S1. Alleles are summarized in Supplementary Table S2 and Transgenes were made by microinjection following standard protocol^71^ and are summarized in Supplementary Table S3. Cloning primers and genotyping primers are summarized in Supplementary Table S4 and S5, respectively. The *juIs559* integrated transcriptional reporter of *sek-1* was produced from CZ24469: *juEx7488* following standard UV/TMP mutagenesis^72^.

### *nipi-3(0)* suppressor screens

We performed two screens to isolate mutations that suppress lethality of *nipi-3(0).* One screen was conducted using visual isolation of suppressors of CZ22446: *nipi-3(ju1293); juEx6807[nipi-3(*+*); Pmyo-2::mCherry]* as previously described^6^. Briefly, suppressors were selected based on ability to survive to adulthood without expression of the extrachromosomal NIPI-3 rescue array, as indicated by the expression of the co-injection marker, *Pmyo-2::mCherry* (Addgene plasmid #19327; http://n2t.net/addgene:19327; RRID:Addgene_19327)*,* and confirmed by genotyping (Supplementary Fig. S1A). Previously, we reported 7 suppressor mutations covering 5 genes^6^. Two additional suppressor alleles, *ju1369* and *ju1371* from this screen are reported here.

In the second suppressor screen, we designed a PEEL-induced selection scheme (Supplementary Fig. S1B)^14^. Briefly, we mutagenized L4 animals of CZ24853: *nipi-3(ju1293); juEx7152[nipi-3(*+*); Phsp::peel-1; Pmyo-2::GFP]* using ethyl methane sulphonate (EMS, three rounds of mutagenesis with concentration between 25 and 40 mM) following a standard protocol. After mutagenesis, L4 animals were placed on seeded NGM plates, approximately 1000 P0s were screened. The F_2_ progeny were subjected to a two-hour heat shock at 37 °C to induce expression of the toxic protein PEEL-1 from *Phsp:*:*peel-1*, which results in killing animals expressing *juEx7152.* We define suppressor mutations as those that can reverse the developmental arrest phenotype of *nipi-3(0)* such that the homozygous suppressor mutations with *nipi-3(0)* propagate for generations, in the absence of *juEx7152*. To ensure independent isolation of suppressor mutations, we kept only one suppressor per P0 plate, which led to total 35 isolates. Sanger sequencing revealed 15 of 35 isolates are mutations within *cebp-1* and 13 of 35 isolates, within *mak-2* (Supplementary Fig. S1C). Additionally, whole genome sequencing revealed 6 of 7 remaining isolates are mutations within *tir-1, nsy-1,* or *sek-1* (Supplementary Figs. S1D and S1E). Whole genome sequencing and recombinant mapping of one remaining isolate, *ju1541*, revealed that the mutation is likely on Chromosome X between − 9.17 and − 2.92 mu, but has not yet been fully mapped (Supplementary Fig. S1E).

### Outcrossing

For *ju1369*, the first two outcrosses were done using CZ10175: *zdIs5[Pmec-4::GFP]* (RRID:WB-STRAIN:WBStrain00005421), which helped identify cross-progeny. The next two outcrosses were done with N2 males and cross progeny were selected for wild-type phenotype. *nipi-3(ju1293); ju1369* was visibly dumpy and egg laying defective.

For *ju1371,* the first outcross was done with CZ10969: *muIs32[Pmec-7::GFP],* which helped to identify cross progeny. The second outcross was done with CZ10175: *zdIs5[Pmec-4::GFP]*. The next two outcrosses were done with N2 males and cross progeny were selected for wild-type phenotype. *nipi-3(ju1293); ju1371* was visibly dumpy and egg laying defective.

For the suppressor strains isolated in the screen with selection, the first outcrossing was done with CZ10175: *zdIs5[Pmec-4::GFP]*. All subsequent outcrossing was done using CZ22446: *nipi-3(ju1293); juEx6807[nipi-3(*+*); Pmyo-2::mCherry]*, which both allowed for identification of cross progeny and replacement of the X chromosome.

### Whole-genome sequencing analysis

Genomic DNA was prepared using the Puregene Cell and Tissue Kit (Qiagen) according to the manufacturer’s instructions and 20 × coverage of sequences was obtained using a 90 bp paired-end Illumina HiSeq 2000 at Beijing Genomics Institute. The raw sequence reads were mapped to the *C. elegans* reference genome (ce10) using Burrows-Wheeler Aligner^73^ in the Galaxy platform (http://use-galaxy.org)^74^ followed by a custom workflow incorporating tools including SAMtools^75^, Genome Analysis Toolkit (GATK), SnpSift, and SnpEff.

The whole genome sequence data set of CZ23399: *nipi-3(ju1293); ju1369* (0 × outcross from EMS isolate) was compared to the whole genome sequencing data obtained of the parental strain, CZ22446: *nipi-3(ju1293); juEx6807[nipi-3(*+*)]* and the whole genome sequencing data from the other strains isolated in this screen. Candidate SNPs were identified by being unique to only the data from CZ23399 and predicted to alter the function of the protein*.* Candidate SNPs were verified by Sanger sequencing, and used to track chromosomal recombinants over the course of four outcrosses as previously described^76^. This analysis placed *ju1369* on Chromosome V between − 8 and + 6 mu. Inspection of mutagenesis induced SNPs in this region revealed a missense mutation (449 g>a, G150E) in the N-Acetyltransferase domain of *natc-2* (Fig. 1A)*.*

Whole genome sequencing data for CZ23401: *nipi-3(ju1293); ju1371* (0 × outcross from EMS isolate) and CZ23692: *nipi-3(ju1293) ; ju1371* (2 × outcross from EMS isolate) was analyzed to guide further mapping as previously described^76^. The whole genome sequence data set of CZ23401 and CZ23692 was compared to the whole genome sequencing data obtained of the parental strain, CZ22446: *nipi-3(ju1293); juEx6807[nipi-3(*+*)]*. Candidate SNPs were identified by present in both the non-outcrossed and two times outcrossed isolates of *ju1371* and not present in the parental strain. Candidate SNPs were tracked by Sanger sequencing over the course of two additional outcrosses, a total of four times outcrossed from the original EMS isolate. During this outcrossing, the *nipi-3(0)* phenotype was never re-isolated, indicating that the suppressor was likely linked to *nipi-3.* Mapping of candidate SNPs and the likely linkage to *nipi-3* placed *ju1371* on Chromosome X to the right of + 12 mu. Inspection of mutagenesis induced SNPs in this region revealed a missense mutation (1817 g>a, G606D) in *hda-4* (Fig. 3A)*.*

Reads from CZ25728, CZ25732, CZ25734, CZ25738, CZ25740, and CZ25742 were searched for mutations within previously identified suppressors of *nipi-3(0).* All strains contained nonsense, missense, or splice site mutations in previously identified suppressors of *nipi-3(0)* (Supplementary Fig. S1D).

### CRISPR-Cas9-mediated genome editing

We generated *hda-4(ju1403), natc-2(ju1797)*, *natc-2(ju1866)*, *natc-3(ju1837)* deletion alleles using the co-CRISPR method^77,78^. CRISPR RNAs (crRNA) sequences are summarized in Supplementary Table S6 and were ordered from Integrated DNA Technologies. For each gene, two crRNAs were used, one targeting the 5’ region of the gene and one targeting the 3’ region. A mixture of gene-specific crRNA (for *hda-4*, *natc-2,* or *natc-3,* respectively, at 0.3 µL of 200 µM), *dpy-10* crRNA (0.3 µL of 200 µM), tracrRNA (0.9 µL of 100 µM), and Cas9 protein (3.5 µL of 40 µM; MacroLabs, University of California, Berkeley) was injected into N2 for *ju1403* and *ju1837,* WU1036: *natc-1(am138)* for *ju1797,* or CZ27728: *nipi-3(0); juIs559[Psek-1::GFP]; juEx6807[nipi-3(*+*)]* for *ju1866*. F_1_ animals displaying dumpy and/or roller phenotype were single housed on NGM plates and propagated to the F_2_ generation. Animals containing large deletions based on the size of the PCR of the targeted gene were isolated and subsequent generations were tracked to confirm homozygosity. Sanger sequencing was used to identify the bounds of the deletion. Strains were outcrossed with N2 to remove dumpy phenotype and maintain desired deletion.

GFP knock-in (KI) at the *natc-1, natc-2,* and *mak-2* loci were produced by standard methods^79^. We designed sgRNAs: agctttacgggtccaatgcc for *natc-1,* ttgccataatgaaagagtac for *natc-2*, and aaagccataatttcgtccgg for *mak-2*. A mixture of 20 ng/µl of sgRNA, 80 ng/µl of homology arm repair template, and 2.5 ng/µl of pCFJ90 *Pmyo-2::mCherry* (Addgene plasmid 19327) was injected into N2 animals. 3 days after injection, hygromycin was added to the plates to kill the untransformed F_1_ animals. On day 6 post-injection, we looked for candidate GFP^KI^ animals, which were L4/adult roller, survived hygromycin selection and without the mCherry extrachromosomal array. We then heat shocked 20 L1/L2 candidate KI worms at 34 °C for 4 h to remove the self-excising cassette. Non-roller animals were checked for GFP expression using compound microscopy and GFP genomic insertion was confirmed by genotyping PCR and sequencing. The sequence of the bounds of the GFP insertion are in Supplementary Fig. S2.

### Body length measurement and animal staging

To quantify the developmental arrest defect caused by *nipi-3* deficiency, we examined body length 72 h after egg-laying in each suppressor mutant in the *nipi-3(0)* background. We also counted the number of animals that reached specific developmental stages based on the presence of embryos as well as completed vulval formation and found that the body length is generally correlated with the percentage of animals that reached L4 or adult stages. The body length, thereby, is primarily chosen to quantify the suppression of *nipi-3(0)* developmental defects.

For quantification of body length, all strains were maintained at 20 °C under well-fed conditions for at least three generations before imaging. Animals were synchronized by placing 20–40 gravid adults on a seeded NGM plate to lay eggs for 2 h and imaged 72 h after egg-laying. Images were acquired on a Zeiss Axioplan compound microscope on 2% agarose pads anesthetized with 2.5 mM levamisole. At least 30 animals were imaged for each genotype. For all strains containing the *nipi-3* rescue array (*juEx6807)*, the gravid adults were array positive. Quantification of body length was done using ImageJ software (RRID:SCR_003070) by manually drawing a segmented line along the length of the animal and the developmental stage of the animal was recorded. Both the imaging and quantification were done under genotype-blind conditions.

### Fluorescence microscopy and imaging

Images were taken on a Zeiss LSM 800 confocal microscope using the 10 × and 63 × objectives. Animals were mounted on 2% agarose pads anesthetized in 2.5 mM levamisole. All strains were maintained at 20ºC under well-fed conditions for at least three generations before imaging.

For quantification of *Psek-1::GFP* intensity, animals with *juIs559[Psek-1::GFP]* reporter were synchronized by placing 20–40 gravid adults on a seeded NGM plate and allowing them to lay eggs for 2 h. Ten animals of a single genotype, 36 h post egg-laying, were aligned on 2% agarose pads anesthetized in 25 mM sodium azide. Images were acquired on a Zeiss Axioplan compound microscope using 10 × objective and 850 ms exposure time across all genotypes. Microscope settings were such that no pixels were saturated in any images. This protocol was repeated over three days to collect images of 30 animals. Quantification of expression of *juIs559* reporter was done using ImageJ. Although there was a drastic change in GFP intensity throughout the animal in multiple tissues, we quantified the anterior region of the head to avoid auto-fluorescence from the intestine. The nerve ring was not included in the analysis of region of interest (ROI) because there was very high expression in the nerve ring in all genetic backgrounds. Images were analyzed by drawing an elliptical ROI in the head of each animal, anterior to the nerve ring, and measuring mean intensity. The image in Fig. 4H is a single slice from a confocal microscope to show a high magnification of the region analyzed. The background was subtracted from each of the readings. The background was quantified by the average of the mean intensity of four elliptical ROIs, each placed near the heads of the animals where no animals are present. Both the imaging and quantification of the *juIs559* reporter were done under genotype-blind conditions.

For imaging strains with *wgIs563[Pcebp-1::CEBP-1::GFP]*^59^ or GFP^KI^ animals were synchronized by moving eggs to an NGM plate and picking just-hatched animals to a fresh seeded NGM plate. At the designated stages these animals were imaged on 10% agar pads anesthetized with 0.25 mM levamisole. Quantification of expression of NATC-1 GFP^KI^ and NATC-2 GFP^KI^ was done using ImageJ. We quantified the fluorescence in the cytoplasm of intestine, the epidermis, and the gonad regions. Images were analyzed by drawing a 10–25 circular ROIs in the tissue of interest and measuring mean intensity. In the intestine, the ROIs were placed next to the intestinal lumen, avoiding any dark areas (cell nuclei) or gut granules. In the epidermis, the focal plane included the nuclei of the seam cells and the ROIs were placed on either side of the seam cells, avoiding cell nuclei. In the gonad, the ROIs were placed between the nuclei, as identified as dark circles. Figure 2 are representative of the images that were analyzed. The background was subtracted from each of the readings. The background of each image was quantified by the average of the mean intensity of four circular ROIs placed where no animals are present. The quantification of the NATC-1 GFP^KI^ and NATC-2 GFP^KI^ strains was done under genotype-blind conditions.

### Approval for animal experiments

We primarily used *C. elegans* as research organisms, which do not require animal protocols. No live vertebrates or higher invertebrates were involved for this study.

## Supplementary Information

Supplementary Information.

## Data Availability

Strains are available from the corresponding author, YJ, upon reasonable request.
