# Supplementary material for: Forward genetic screening identifies novel roles for N-terminal acetyltransferase C and histone deacetylase in C. elegans development
Source: Sci Rep. 2022 Sep 30;12:16438. doi: 10.1038/s41598-022-20361-x (PMC9525577; doi:10.1038/s41598-022-20361-x)
Supplement: Supplementary file 1 — Supplementary Information. [file 41598_2022_20361_MOESM1_ESM.pdf]

Scientific Reports

Supplementary Information for

Forward Genetic Screening Identifies Novel Roles for N-terminal Acetyltransferase C and Histone Deacetylase in *C. elegans* Development

Rose Aria Malinow, Ming Zhu, Yishi Jin* and Kyung Won Kim*

*Corresponding authors: Yishi Jin (yijin@ucsd.edu) and Kyung Won Kim (kwkim@hallym.ac.kr)

This PDF file includes:

Supplementary Text

Figures S1 to S5

Tables S1 to S6

References

Malinow *et al.*
Figure S1

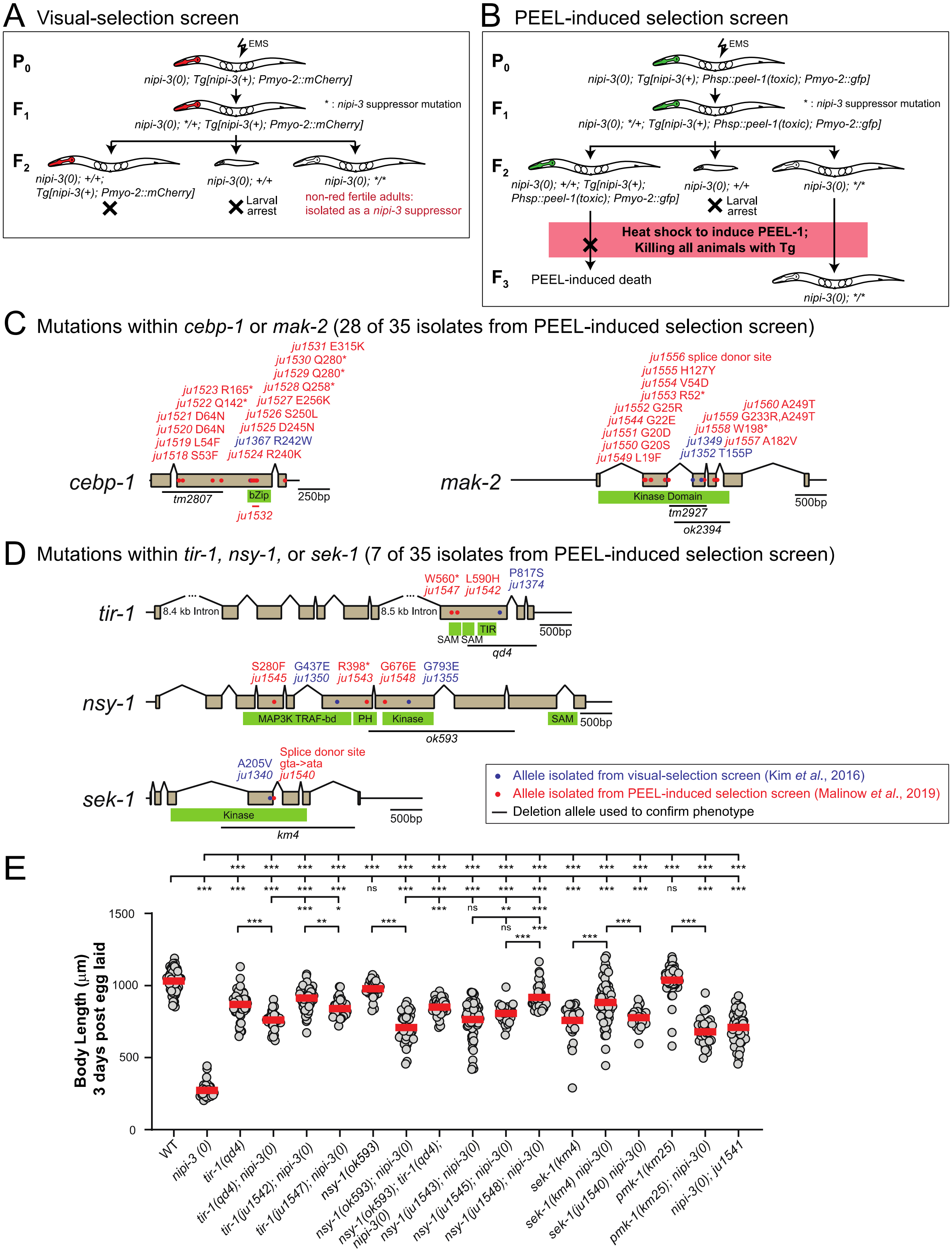

Supplementary Figure S1. Forward genetic screens designed to identify the suppressors of *nipi-3(0)* larval arrest and lethality

(A) Schematic overview of the visual-selection screen (adapted from Kim *et al*., 2016). Non-fluorescent animals at F_2 generation were selected as suppressors of *nipi-3(0).*

(B) Schematic overview of the screen relying on selection against peel-induced toxicity. Mutagenized animals contain a transgene (Tg) containing WT *nipi-3*, the gene encoding a toxic protein, PEEL-1, under a heat shock (*hsp)* promoter, and a fluorescent co-injection marker that expresses GFP under the pharyngeal muscle (*myo-2)* promoter. The mutagenized P0 animals were grown on seeded NGM plates to F_2 generation without transferring any animals to fresh plates. The plates were subjected to 1 hour heat shock at 37ºC (illustrated by red box) to induce expression of PEEL-1, effectively killing all animals containing the transgene. The only surviving animals contain a mutation that suppresses the larval arrest of *nipi-3(0).* For the first round of screening, 100 L4 animals were isolated on individual seeded NGM plates. Of these 100 P0 plates, seven had animals that survived selection. For the next two rounds of mutagenesis, to increase the coverage of our screen and to minimize the efforts to set up the screen, we no longer singled individual P0s onto their own plates. Instead, a mixed population of about 20-30 animals, containing mostly L4s, was placed together on a seeded NGM plate. We later found that this protocol biased the screen towards isolation of suppressors that have a large brood size and can recover from starvation. In addition to any strong suppressor being able to out-compete a suppressor that simply rescued viability and fertility, but did not have a large brood size, many of the plates ran out of food as the animals reached F_2 stage. We later discovered that rescue of *nipi-3(0)* larval arrest is not always linked to the ability to recover from starvation.

(C–D) Mutations within **(D)** *cebp-1* or *mak-2* **(D)** *tir-1, nsy-1,* or *sek-1.* Illustrations of gene structures, missense alleles, and deletion alleles. Exons are tan boxes, introns are angled black lines connecting exons, and the UTRs are black horizonal lines. Protein motifs are illustrated in green. Missense alleles isolated in EMS screen for suppressors of *nipi-3(0)* larval arrest are indicated as a dot in the exon where the mutation exists, allele name and amino acid substitution are labeled above the dot. Blue dots indicate the allele was isolated and previously published [1] and red dots indicate novel alleles from this screen. Deletion alleles are indicated as black or red horizonal lines showing the region of DNA that is deleted.

(E) Quantification of body length of animals 3 days post egg laid. *nipi-3(0)* mutants have a short body length. This phenotype is rescued by loss of function or deletion mutations of *tir-1, nsy-1,* and *sek-1.* Different alleles within the same gene can have significantly different suppression of *nipi-3(0)* body length. This may be caused by different effects on the expression and function of the protein. mRNA was not collected to confirm expression of these mutant proteins. Each dot represents a single animal, each red line represents the mean value. Statistics: One-Way ANOVA with Tukey's post hoc test. *ns* not significant, *P<0.05, **P<0.01, ***P<0.001.

A

```
C.elegans NATC-2     ------------------DIMRLITKDLSEPYSIYTYRYFLHNWPEYCFLAYDQTNNTYI G   150
H.sapiens Naa30      EDRTIRYVRYESELQMPDIMRLITKDLSEPYSIYTYRYFIHNWPQLCFLAMV--GEECV G   266
                                       ***********************:****: ****    .:  :*

C.elegans NATC-2     AVLCKLELDMYGRCKGYLAM LAVDESCRRLGI GTRLVRRALDAMQSKGCDEIVLETEVSN   210
H.sapiens Naa30      AIVCKLDMHKKMFRRGYIAM LAVDSKYRRNGI GTNLVKKAIYAMVEGDCDEVVLETEITN   326
                     *::***::.     :**:******.. ** ****.**::*: ** . .***:*****::*

C.elegans NATC-2     KNAQRLYSNLGFIRQKRLLKYYLNGGDAF-------   239
H.sapiens Naa30      KSALKLYENLGFVRDKRLFRYYLNGVDALRLKLWLR   362
                     *.* :**.****:*:***::***** **:
```

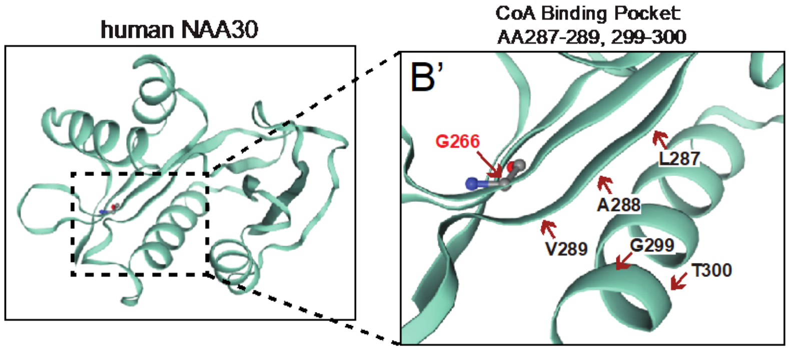

C

```
C.elegans NATC-3     ---------------MCDDQSTDL--IQLKGH-ENAKKSEAHLKVLEYIGKR YEIRMTDG   42
H.sapiens Naa38      MAGAGPTMLLREENGCCSRRQSSSSAGDSDGERED SAAERARQQLEALLNKT MRIRMTDG   60
                                     *. :.:.     : .*. *::   ..*: ::    :.*  .******

C.elegans NATC-3     RYIRGTMIATDKDANMVFN KADERWDKDPQL--KGVRFLG QAMISKKHVESMHALPDPKE   100
H.sapiens Naa38      RTLVGCFLCTDRDCNVILG SAQEFLKPSDSFSAGEPRVLG LAMVPGHHIVSIEVQRESLT   120
                      *  :  * ::.**:*.*::::..*:*  .  . .:     *.** **:  :*: *:..  :

C.elegans NATC-3     TEI--     103
H.sapiens Naa38      GPPYL     125
```

Supplementary Figure S2. NATC-2 and NATC-3 is homologs to hNaa30 and hNaa38, respectively.

(A) Protein alignment of the functional domain (RimI) of *C. elegans* NATC-2 and human NAA30. The domain is 62% identical and 76% similar and spans amino acids 108-239 in NATC-2 and amino acids 209-362 in hNAA30. The CoA binding pocket, indicated in blue text, has 100% identity between the human and *C. elegans* proteins. The mutated residue in *ju1369* is indicated in red text.

(B) Crystal structure of hNAA30, homologue of NATC-2 **(B')** Inset shows CoA binding pocket and the location of the mutated residue in *ju1369* (G150E mutation). *C. elegans* G150 is homologous to human G266. Thus, *natc-2(ju1369)* is predicted to be adjacent to CoA binding pocket.

(C) Protein alignment of *C. elegans* NATC-3 (Y38G1C.9) and human Naa38, the auxiliary subunit of the human NatC complex. The proteins are 25% identical and 41% similar. The functional domains (Sm1, Sm2), indicated in bold, have 41% identity and 80% similarity between the human and *C. elegans* proteins.

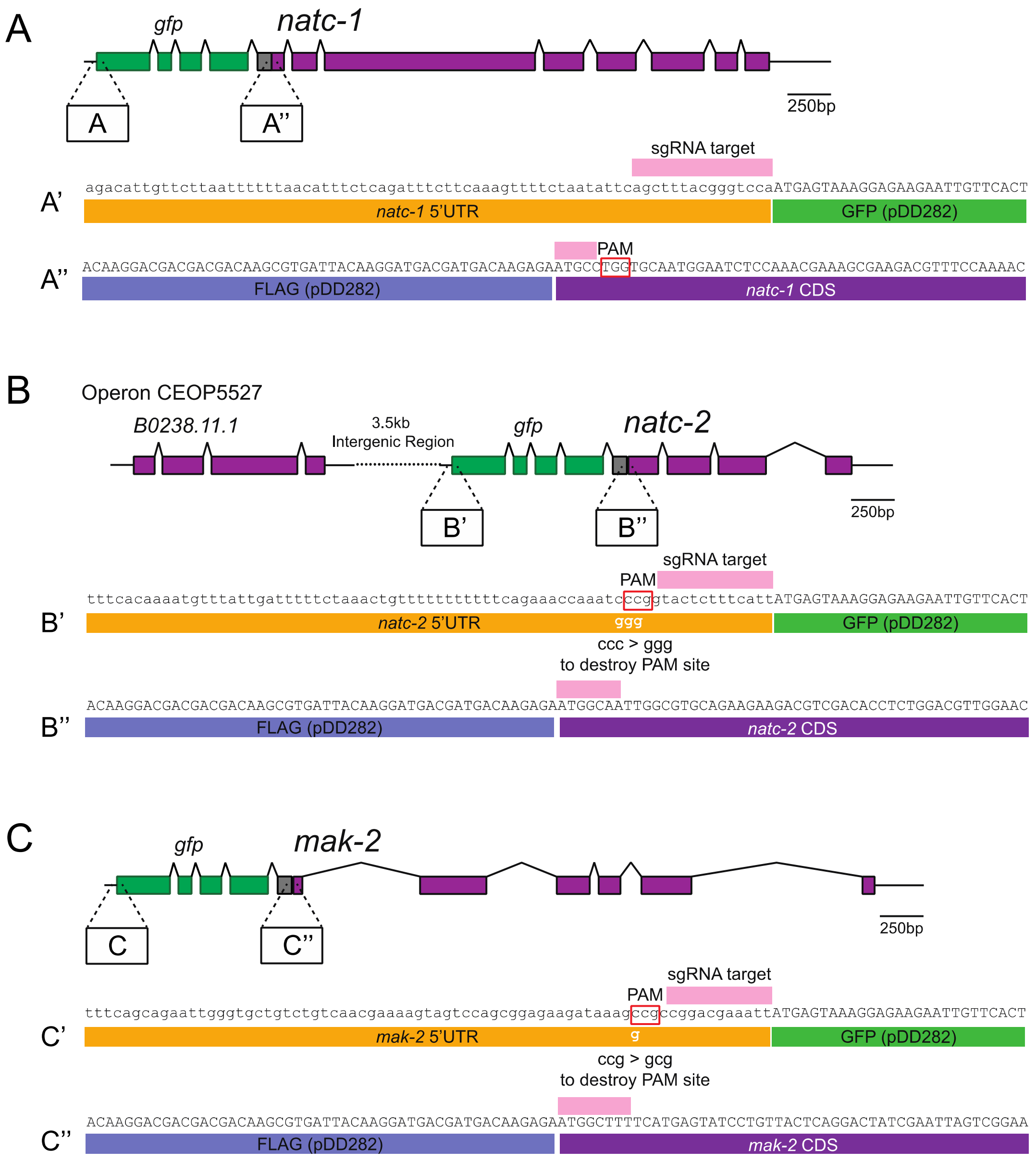

Supplementary Figure S3. GFP knock-in sequence for *natc-1, natc-2,* and *mak-2*

(A–C) GFP knock-in sequence for **(A)** *natc-1* **(B)** *natc-2*, and **(C)** *mak-2*. Illustration of gene structure and GFP::3xFLAG insertion after removal of self-excision cassette. Exons are purple boxes; introns are angled black or green lines connecting exons; and GFP exons are green boxes. **(A', B', C')** Sequence of each gene's 5'UTR and the beginning of the GFP::3xFLAG insertion from pDD282. The targeting sequence for the sgRNA is illustrated in pink. **(A'', B'', C'')** Sequence of the end of the GFP::3xFLAG insertion and the beginning of the coding sequence for each gene. The targeting sequence for the sgRNA is illustrated in pink and the PAM site is outlined in red. In the primer used to amplify the 5' arm for the repair template plasmid, a silent mutation was introduced to mutate the PAM site and prevent Cas9 cutting again after the insertion of the GFP construct.

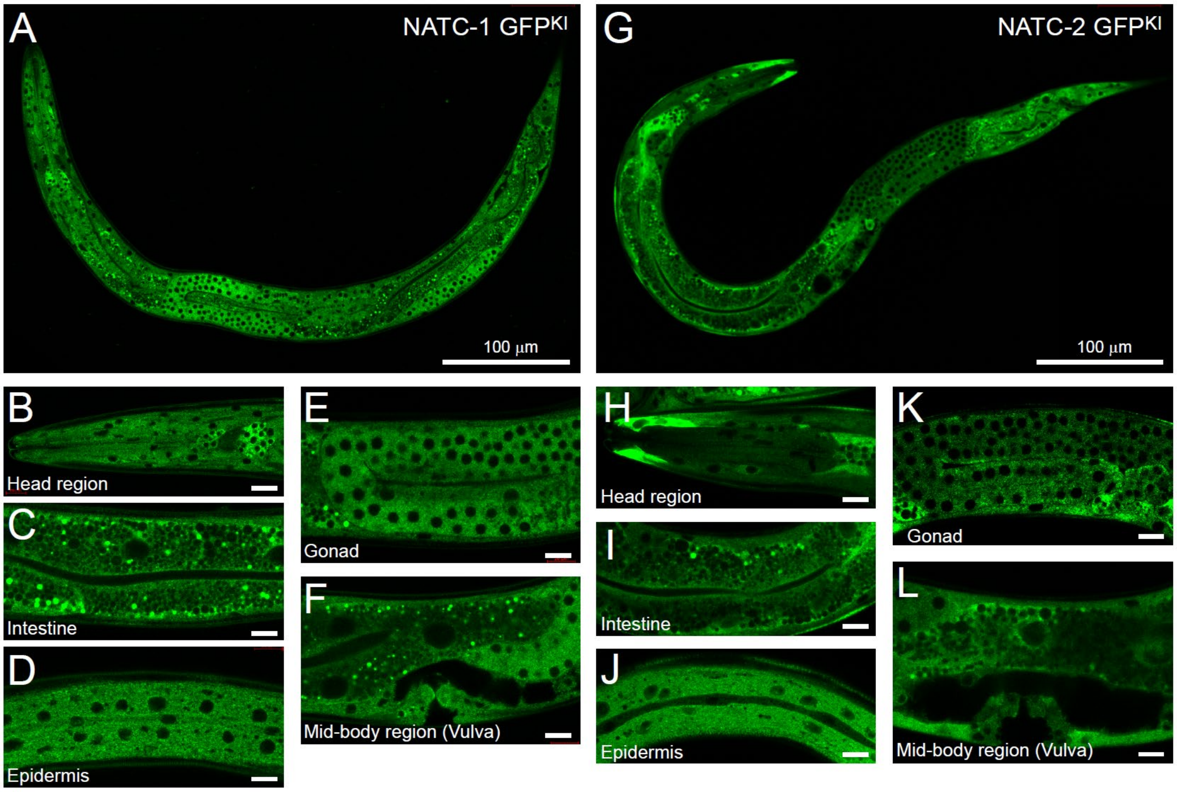

Supplementary Figure S4. NATC-1 and NATC-2 are expressed in many tissues in *C. elegans.*

(A) Single plane confocal images of NATC-1 GFP^{KI} (*ju1801*) in young adult animals. This reporter shows N' tagged NATC-1 protein is expressed throughout the animals including **(B)** head region, **(C)** intestine, **(D)** epidermis, **(E)** gonad, and **(F)** mid-body region (vulva). **(G)** Single plane confocal images of NATC-2 GFP^{KI} (*ju1803*) in young adult animals. This reporter shows N' tagged NATC-2 protein is expressed throughout the animals including **(H)** head region, **(I)** intestine, **(J)** epidermis, **(K)** gonad, and **(L)** mid-body region (vulva). This tagged protein is functional, based on phenotype when combined with *nipi-3(0)*. Images have been adjusted for better visualization. Scale bars A and G = 100 µm, Scale Bars B–F and H–L = 10 µm.

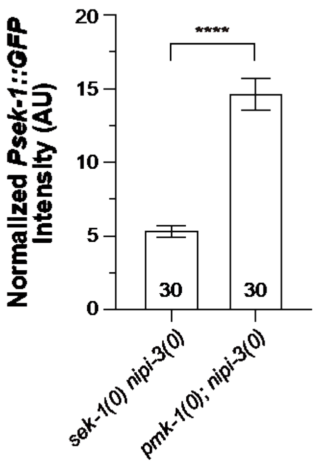

Supplementary Figure S5. ***pmk-1(0)*** **has a significantly weaker effect on** ***sek-1*** **transcription than** ***sek-1(0).***

Quantification of *Psek-1::GFP* expression in animals 36 hours post egg laid. Data are represented as mean ± SEM. n, number of animals shown within columns Statistics: Mann–Whitney test. ****P < 0.0001.

Supplementary Table S1. Strains and Genotypes

Strain	Genotype
CZ23399[#]	*natc-2(ju1369) V; nipi-3(ju1293) X (EMS isolate)*
CZ22446[#]	*nipi-3(ju1293) X; nipi-3 gDNA(juEx6807) (pre-mutagenized strain)*
CZ28337	*natc-3(ju1837) I; nipi-3(ju1293) X; Psek-1::GFP(juIs559); nipi-3 gDNA(juEx6807)*
N2	*wild type*
CZ24194	*natc-2(ju1369) V; nipi-3(ju1293) X*
CZ27143	*natc-1(ok2062) V; nipi-3(ju1293) X; nipi-3 gDNA(juEx6807)*
CZ28553	*natc-3(ju1837) I; natc-2(ju1797) V natc-1(am138) V; nipi-3(ju1293) X; nipi-3 gDNA(juEx6807)*
CZ28664	*natc-2(ju1866) V; nipi-3(ju1371) X; Psek-1::GFP(juIs559) ; nipi-3 gDNA(juEx6807)*
CZ28362	*natc-1(ok2062) V; nipi-3(ju1293) X; Psek-1::GFP(juIs559); nipi-3 gDNA(juEx6807)*
CZ27861	*natc-2(ju1797) V natc-1(am138) V; nipi-3(ju1293) X; Psek-1::GFP(juIs559); nipi-3 gDNA(juEx6807)*
CZ28028	*GFP::natc-1(ju1801) V; nipi-3(ju1293) X; nipi-3 gDNA(juEx6807)*
CZ28029	*GFP::natc-2(ju1803) V; nipi-3(ju1293) X; nipi-3 gDNA(juEx6807)*
CZ23401[#]	*nipi-3(ju1293) X hda-4(ju1371) X (EMS isolate)*
CZ23692[#]	*nipi-3(ju1293) X hda-4(ju1371) X (2x O.C.)*
CZ24455	*nipi-3(ju1293) X hda-4(ju1371) X; Phda-4::hda-4(genomic)::gfp(oyIs73); nipi-3 gDNA(juEx6807)*
CZ24911	*nipi-3(ju1293) X hda-4(ju1371) X Pcebp-1(2.2kb): flag::cebp-1::cebp-1 3'UTR(juIs418) X ; nipi-3 gDNA(juEx7233)*
CZ24195	*nipi-3(ju1293) X hda-4(ju1371) X*
CZ28249	*hda-4(ju1371) X; Psek-1::GFP(juIs559)*
CZ27919	*hda-4(ju1403) X; Psek-1::GFP(juIs559)*
CZ22882	*mef-2(gv1) I; nipi-3(ju1293) X; nipi-3 gDNA(juEx6807)*
CZ28030	*mef-2(gv1) I; nipi-3(ju1293) X hda-4(ju1371) X; nipi-3 gDNA(juEx6807)*
CZ28606	*nipi-3(ju1293) X hda-4(ju1371) X; cebp-1::GFP(wgIs563); nipi-3 gDNA(juEx6807)*
CZ24959	*Psek-1(∆CEBP-1 binding sites)::gfp(juEx7617)*
CZ24961	*nipi-3(ju1293) X; nipi-3 gDNA(juEx6807); Psek-1(∆CEBP-1 binding sites)::gfp(juEx7617)*
CZ27584	*Psek-1::GFP(juIs559)*
CZ27809	*cebp-1(tm2807) X; Psek-1::GFP(juIs559)*
CZ27728	*nipi-3(ju1293) X; Psek-1::GFP(juIs559); nipi-3 gDNA(juEx6807)*
CZ27585	*Psek-1::GFP(juIs559)*
CZ27864	*cebp-1(tm2807) X nipi-3(ju1293) X; Psek-1::GFP(juIs559); nipi-3 gDNA(juEx6807)*
CZ28038	*tir-1(qd4) III; nipi-3(ju1293) X; Psek-1::GFP(juIs559); nipi-3 gDNA(juEx6807)*
CZ28370	*sek-1(km4) nipi-3(ju1293); Psek-1::GFP(juIs559)*
CZ27136	*pmk-1(km25) IV; nipi-3(ju1293) X; Psek-1::GFP(juIs559); nipi-3 gDNA(juEx6807)*
CZ28037	*nsy-1(ok593) II; nipi-3(ju1293) X; Psek-1::GFP(juIs559); nipi-3 gDNA(juEx6807)*
CZ28160	*nsy-1(ok593) II; mak-2(ok2394) IV; Psek-1::GFP(juIs559)*
CZ25830	*cebp-1::GFP(wgIs563)*
CZ24854	*nipi-3(ju1293) X; cebp-1::GFP(wgIs563); nipi-3 gDNA(juEx6807)*
CZ28459	*mak-2(tm2927) IV; nipi-3(ju1293) X; cebp-1::GFP(wgIs563); nipi-3 gDNA(juEx6807)*
CZ27898	*GFP::natc-1(ju1801) V*
CZ27900	*GFP::natc-2(ju1803) V*
CZ28637	*GFP::SEC::mak-2(ju1850) IV*
CZ28638	*GFP::mak-2(ju1851) IV*
CZ28605	*GFP::mak-2(ju1851) IV; nipi-3(ju1293) X; nipi-3 gDNA(juEx6807)*
CZ25595	*mak-2(ju1544) IV; nipi-3(ju1293) X*
CZ25600	*mak-2(ju1549) IV; nipi-3(ju1293) X*
CZ25601	*mak-2(ju1550) IV; nipi-3(ju1293) X*
CZ25602	*mak-2(ju1551) IV; nipi-3(ju1293) X*
CZ25603	*mak-2(ju1552) IV; nipi-3(ju1293) X*
CZ25604	*mak-2(ju1553) IV; nipi-3(ju1293) X*

Strain	Genotype
CZ25605	*mak-2(ju1554) IV; nipi-3(ju1293) X*
CZ25606	*mak-2(ju1555) IV; nipi-3(ju1293) X*
CZ25607	*mak-2(ju1556) IV; nipi-3(ju1293) X*
CZ25608	*mak-2(ju1557) IV; nipi-3(ju1293) X*
CZ25609	*mak-2(ju1558) IV; nipi-3(ju1293) X*
CZ25610	*mak-2(ju1559) IV; nipi-3(ju1293) X*
CZ25611	*mak-2(ju1560) IV; nipi-3(ju1293) X*
CZ25736	*mak-2(ju1544) IV; nipi-3(ju1293) X*
CZ25737	*mec-4-GFP(zdIs5) I; mak-2(ju1544) IV; nipi-3(ju1293) X*
CZ25591	*sek-1(ju1540) X nipi-3(ju1293) X*
CZ25729	*mec-4-GFP(zdIs5) I; sek-1(ju1540) X nipi-3(ju1293) X*
CZ25732[#]	*tir-1(ju1542) III; nipi-3(ju1293) X;*
CZ25733	*mec-4-GFP(zdIs5) I; tir-1(ju1542) III; nipi-3(ju1293) X*
CZ25734[#]	*nsy-1(ju1543) II; nipi-3(ju1293) X*
CZ25735	*mec-4-GFP(zdIs5) I; nsy-1(ju1543) II; nipi-3(ju1293) X*
CZ25738[#]	*nsy-1(ju1545) II; nipi-3(ju1293) X*
CZ25739	*mec-4-GFP(zdIs5) I; nsy-1(ju1545) II; nipi-3(ju1293) X*
CZ25740[#]	*tir-1(ju1547) III; nipi-3(ju1293) X*
CZ25741	*mec-4-GFP(zdIs5) I; tir-1(ju1547) III; nipi-3(ju1293) X*
CZ25742[#]	*nsy-1(ju1548) II; nipi-3(ju1293) X*
CZ25743	*mec-4-GFP(zdIs5) I; nsy-1(ju1548) II; nipi-3(ju1293) X*
CZ25593	*tir-1(ju1542) III; nipi-3(ju1293) X*
CZ25594	*nsy-1(ju1543) II; nipi-3(ju1293) X*
CZ25596	*nsy-1(ju1545) II; nipi-3(ju1293) X*
CZ25598	*tir-1(ju1547) III; nipi-3(ju1293) X*
CZ25599	*nsy-1(ju1548) II; nipi-3(ju1293) X*
CZ25728[#]	*sek-1(ju1540) X nipi-3(ju1293) X*
CZ24853	*nipi-3(ju1293) X; nipi-3 gDNA + Phsp-peel-1(juEx7152)*
CZ28341	*natc-2(ju1369) V*
CZ8762	*mak-2(ok2394) IV*
WU1036	*natc-1(am138) V*
CZ21653	*sek-1(km4) X*
CZ21739	*tir-1(qd4) III*
CZ27142	*natc-1(am138) V; nipi-3(ju1293) X; nipi-3 gDNA(juEx6807)*
CZ28458	*mak-2(tm2927) IV; nipi-3(ju1293) X; Psek-1::GFP(juIs559); nipi-3 gDNA(juEx6807)*
CZ28552	*mak-2(tm2927) IV; cebp-1(tm2807) X nipi-3(ju1293) X; Psek-1::GFP(juIs559); nipi-3 gDNA(juEx6807)*
CZ23578	*sek-1(km4) X nipi-3(ju1293) X*
CZ27863	*natc-2(ju1797) V natc-1(am138) V; cebp-1(tm2807) X; Psek-1::GFP(juIs559)*
CZ27917	*mak-2(ok2394) IV; Psek-1::GFP(juIs559)*
CZ28159	*nsy-1(ok593) II; cebp-1(tm2807) X; Psek-1::GFP(juIs559)*
CZ28369	*sek-1(km4) X; Psek-1::GFP(juIs559)*
CZ28373	*tir-1(qd4) III; Psek-1::GFP(juIs559)*
CZ28374	*nsy-1(ok593) II; tir-1(qd4) III; Psek-1::GFP(juIs559)*
CZ27727	*natc-2(ju1797) V natc-1(am138) V; Psek-1::GFP(juIs559)*
CZ28354	*natc-3(ju1837) I; Psek-1::GFP(juIs559)*
CZ28361	*natc-1(ok2062) V; Psek-1::GFP(juIs559)*
CZ28363	*natc-3(ju1837) I; natc-1(am138) V; Psek-1::GFP(juIs559)*
CZ27135	*pmk-1(km25) IV; Psek-1::GFP(juIs559)*
CZ28372	*nsy-1(ok593) II; Psek-1::GFP(juIs559)*

Strain	Genotype
CZ27862	*natc-2(ju1797) V natc-1(am138) V; cebp-1(tm2807) X nipi-3(ju1293) X; Psek-1::GFP(juIs559); nipi-3 gDNA(juEx6807)*
CZ28140	*cebp-1(tm2807) X nipi-3(ju1293) X hda-4(ju1371) X; nipi-3 gDNA(juEx6807)*
CZ28161	*nsy-1(ok593) II; mak-2(ok2394) IV; nipi-3(ju1293) X; Psek-1::GFP(juIs559) ; nipi-3 gDNA(juEx6807)*
CZ28181	*nipi-3(ju1293) X hda-4(ju1403) X; Psek-1::GFP(juIs559)*
CZ28142	*nipi-3(ju1293) X hda-4(ju1371) X; Psek-1::GFP(juIs559); nipi-3 gDNA(juEx6807)*
CZ27918	*mak-2(ok2394) IV; nipi-3(ju1293) X; Psek-1::GFP(juIs559); nipi-3 gDNA(juEx6807)*
CZ28036	*mak-2(ok2394) IV pmk-1(km25) IV; nipi-3(ju1293) X; Psek-1::GFP(juIs559); nipi-3 gDNA(juEx6807)*
CZ28371	*nsy-1(ok593) II; cebp-1(tm2807) X nipi-3(ju1293) X; Psek-1::GFP(juIs559)*
CZ28035	*mak-2(ok2394) IV; cebp-1(tm2807) X nipi-3(ju1293) X; Psek-1::GFP(juIs559); nipi-3 gDNA(juEx6807)*
CZ28364	*natc-3(ju1837) I; natc-1(am138) V; nipi-3(ju1293) X; Psek-1::GFP(juIs559); nipi-3 gDNA(juEx6807)*
CZ28039	*nsy-1(ok593) II; tir-1(qd4) III; nipi-3(ju1293) X; Psek-1::GFP(juIs559); nipi-3 gDNA(juEx6807)*

[#] - strains analyzed by whole genome sequencing

Supplementary Table S2. Alleles

Allele	Gene	Effect	Flanking Sequence	Detection
am138	*natc-1*	Deletion	ccaaacgaaagcgaa/ /acacattaaaacatt	PCR: YJ12710 + YJ12711<br>WT: 429 mut: 243
ju1293	*nipi-3*	Deletion	ttatctcatttgctt/ /atccagctccgcacc	PCR with Platinum II 2xMM Taq:<br>YJ11161 + YJ12708<br>WT: 990 mut: 400
ju1369	*natc-2*	G150E	acctacatcgAagctgttttg	PCR: YJ12701 + YJ12715<br>Sequence: YJ12701
ju1371	*hda-4*	G606D	cggccgccagAtcatcatgca	PCR: YJ12700 +YJ12709<br>Sequence: YJ12700
ju1403	*hda-4*	Deletion	ctcgccctgcccctg/ /aaccgttattgagcc	PCR: YJ12707 +YJ12700 +YJ12709<br>WT: 670 mut: 390
ju1540	*sek-1*	splice site	catggccaagAtacggaaaat	PCR: YJ12702 +YJ12695<br>Digest: StyI<br>WT: 174/87 mut: 261
ju1542	*tir-1*	L590H	gatggagatcAtttacttcaa	PCR: YJ12719 + YJ12699R<br>Sequence: YJ12719
ju1543	*nsy-1*	R598*	tgaatcgaaaTgagatgatcg	PCR: YJ12720 + YJ12721<br>Sequence: YJ12720
ju1544	*mak-2*	G22E	ctcggcgtagAaatcaacgga	PCR: YJ12690 + YJ12691<br>Sequence: YJ12690
ju1545	*nsy-1*	S280F	gataccgtatTccttatgatg	PCR: YJ12703 + YJ12704<br>Sequence: YJ12703
ju1547	*tir-1*	W560*	gtacctggctAgacatgcgca	PCR: YJ12719 + YJ12699R<br>Sequence: YJ12719
ju1548	*nsy-1*	G676E	ggaacctatgAaactgtgtac	PCR: YJ12720 + YJ12721<br>Sequence: YJ12720
ju1549	*mak-2*	L19F	tttacaggttTtcggcgtagg	PCR: YJ12690 + YJ12691<br>Sequence: YJ12690
ju1550	*mak-2*	G20S	acaggttctcAgcgtaggaat	PCR: YJ12690 + YJ12691<br>Sequence: YJ12690
ju1551	*mak-2*	G20D	caggttctcgAcgtaggaatc	PCR: YJ12690 + YJ12691<br>Sequence: YJ12690
ju1552	*mak-2*	G25R	aggaatcaacAgaaaagttgtg	PCR: YJ12690 + YJ12691<br>Sequence: YJ12690
ju1553	*mak-2*	R52*	gaaagctcgtTgagaagtcga	PCR: YJ12690 + YJ12691<br>Sequence: YJ12690
ju1554	*mak-2*	V54D	cgtcgagaagAcgaacttcac	PCR: YJ12690 + YJ12691<br>Sequence: YJ12690
ju1555	*mak-2*	H127Y	tgctcatttgTatcgaatgag	PCR: YJ12690 + YJ12691<br>Sequence: YJ12690
ju1556	*mak-2*	splice site	ttatgtgtgaAttttttagaa	PCR: YJ12690 + YJ12691<br>Sequence: YJ12690
ju1557	*mak-2*	A182V	tactattgtgTtccggaagtg	PCR: YJ9306 + YJ9307<br>Sequence: YJ9306
ju1558	*mak-2*	W198*	gtgatttatgAtcgatcggag	PCR: YJ9306 + YJ9307<br>Sequence: YJ9306
ju1559	*mak-2*	G233R,<br>A249T	aatcaagtcaAgacagtacac,<br>ttctgaagcaAgtaagagtta	PCR: YJ9306 + YJ9307<br>Sequence: YJ9306
ju1560	*mak-2*	A249T	ttctgaagcaAgtaagagtta	PCR: YJ9306 + YJ9307<br>Sequence: YJ9306
ju1797	*natc-2*	Deletion	caacgatattatgcg/ /aatataattttcaag	PCR: YJ12705 + YJ12701 +YJ12715<br>WT: 883 mut: 719
ju1801	*natc-1*	GFP KI	agctttacgggtcca/ /atgcctggtgcaatg	fluorescence microscope
ju1803	*natc-2*	GFP KI	cggtactctttcatt/ /atggcaattggcgtg	fluorescence microscope
ju1837	*natc-3*	Deletion	gaagcccatctgaaa/ /agggcaagcgatgat	PCR: YJ12722 + YJ12723<br>WT: 1799 mut: 751
ju1850	*mak-2*	GFP KI	ccgccggacgaaatt/ /atggcttttcatgag	fluorescence microscope

**Allele**	**Gene**	**Effect**	**Flanking Sequence**	**Detection**
ju1851	*mak-2*	GFP KI	ccgccggacgaaatt/ /atggcttttcatgag	fluorescence microscope
ju1866	*natc-2*	Deletion	gatatgtatggacgg/ /ggaggaaggaactta	PCR: YJ12701F + YJ12706<br>WT: 1408 mut: 736
km4	*sek-1*	Deletion	acactagaataagtg/ /ctatgctagatttgc	PCR: YJ9980F + YJ12717 + YJ12718<br>WT: 508 mut: 830
km25	*pmk-1*	Deletion	atattggttttacgt/ /cgttgtatgtgtcat	PCR: AC2408+ YJ12697 +YJ12698<br>WT: 725 mut: 900
ok593	*nsy-1*	Deletion	gtcaattattcctttt/ /atcacttcagccgtt	PCR: AC2396 + AC2397 + AC2398<br>WT: 851 mut: 587
ok2062	*natc-1*	Deletion	aagcaaaatcggaaa/ /aactttggaacggag	PCR: YJ12712 + YJ12713 + YJ12714<br>WT: 515 mut: 292
ok2394	*mak-2*	Deletion	gaagattgaaagaat/ /tccagatactccatt	PCR: YJ12693 + YJ12692 + YJ12694<br>WT: 435 mut: 784
qd4	*tir-1*	Deletion	ctaccaaatgctcac/ /tttccaacaaaaaac	PCR: YJ10840 + YJ10841 + YJ10842<br>WT: 296 mut: 708
tm2807	*cebp-1*	Deletion	tccagcaatccgagg/ /ttatgacgattacca	PCR: YJ9850 + YJ8980<br>WT: 924 mut: 445
tm2927	*mak-2*	Deletion	acttggctaggaaaa/ /cataaaaatcatata	PCR: YJ12690 + YJ12691 + YJ12692<br>WT: 320 mut: 730

Supplementary Table S3. Transgenes and Plasmids

Transgene	Description	Plasmid(s)
juEx7488	Transcriptional reporter of *sek-1*	5 ng/ul of pKK279 [*Psek-1(4870 bp upstream)::GFP*]<br>90 ng/ul [*Pgcy-8::mCherry*]
juEx7617	Transcriptional reporter of *sek-1* without CEBP-1 binding sites	5 ng/ul of pKK285 [*Psek-1(4870 bp upstream – deletion of 120bp containing two CEBP-1 binding sites)::GFP*]<br>90 ng/ul [*Pgcy-8::mCherry*]
juEx6807	genomic rescue of *nipi-3*	5 ng/ul of pCZGY3044 [*nipi-3 gDNA*]<br>2 ng/ul of pCFJ90 [*Pmyo-2::mCherry*]
juIs559	TMP/UV multicopy integration	pKK279 [*Psek-1(4870 bp upstream)::GFP*]<br>[*Pgcy-8::mCherry*]
wgIs563[#]	Bombardment integration	[*cebp-1::ty1::eGFP::3xFLAG*]<br>[*unc-119(+)*]
oyIs73[##]	Multicopy integration	[*Phda-4::hda-4(genomic)::GFP*]<br>[*Punc-122::RFP*]
juIs418	TMP/UV multicopy integration	pCZGY2508 [*Pcebp-1(2200 bp upstream)::FLAG::CEBP-1::cebp-1 3' UTR*]<br>pCFJ90 [*Pmyo-2::mCherry*]

[#] - source is (Sarov *et al*., 2006)

[##] - source is (van der Linden *et al*., 2007)

Supplementary Table S4. Cloning Primers

Primer	Sequence	Description
YJ12724	acgttgtaaaacgacggccagtcgccggcaatggtgttcctgagccattc	*natc-1* N' GFP^{KI} 5' HA
YJ12725	TCCAGTGAACAATTCTTCTCCTTTACTCATtggacccgtaaagctgaata	
YJ12726	CGTGATTACAAGGATGACGATGACAAGAGAATGCCcGGTGCAATGGAATC	*natc-1* N' GFP^{KI} 3' HA
YJ12727	tcacacaggaaacagctatgaccatgttatGCATGAGCATTTCGAATGAA	
YJ12728	agctttacgggtccaatgccGTTTTAGAGCTAGAAATAGCAAGT	*natc-1* sgRNA into pDD162
YJ12729	acgttgtaaaacgacggccagtcgccggcatcttcgcatttgttgagacg	*natc-2* N' GFP^{KI} 5' HA
YJ12730	TCCAGTGAACAATTCTTCTCCTTTACTCATaatgaaagagtacccccatttgg	
YJ12731	CGTGATTACAAGGATGACGATGACAAGAGAATGGCAATTGGCGTGCAG	*natc-2* N' GFP^{KI} 3' HA
YJ12732	tcacacaggaaacagctatgaccatgttatGCCAACATGGCAAGATAACC	
YJ12733	TTGCCATaatgaaagagtacGTTTTAGAGCTAGAAATAGCAAGT	*natc-2* sgRNA into pDD162
YJ12734	cccagtcacgacgttgtaaaacgacggccagtcgccggcaactctgtgccaccaaaaacc	*mak-2* N' GFP^{KI} 5' HA
YJ12735	GGGACAACTCCAGTGAACAATTCTTCTCCTTTACTCATaatttcgtccggcgCctttatc	
YJ12736	CGACGACAAGCGTGATTACAAGGATGACGATGACAAGAGAATGGCTTTTCATGAGTATCC	*mak-2* N' GFP^{KI} 3' HA
YJ12737	gataacaatttcacacaggaaacagctatgaccatgttataaaagaagcgaaaggtaggc	
YJ12738	AAAGCCATAATTTCGTCCGGGTTTTAGAGCTAGAAATAGCAAGT	*mak-2* sgRNA into pDD162
YJ12739	CCGGACGAAATTATGGCTTTCaagacatctcgcaatagg	
YJ12740	atccattttgctccgatgag	pKK279: *sek-1* promoter
YJ12741	TCGCTCCATgatgtaagactc	
YJ12742	aatgtgtttggtcacaaaaacg	pKK285: remove CEBP-1 binding sites from pKK279
YJ12743	atttagacggtgtgggttcg	

HA = Homology arm to make repair template from pDD282 for GFP knock-in.

pDD162 = a plasmid encoding Cas9 and sgRNA target (Dickinson *et al*., 2015).

Supplementary Table S5. Genotyping Primers

Primer	Sequence	Target Gene
YJ12690	tttgaggtcaggcaggtagg	*mak-2*
YJ12691	cttacgcttaggctgggaac	*mak-2*
YJ9850	ATGACGTCATCATTTCACTTTTC	*cebp-1*
YJ8980	TTAGGTCGGCTCAGCCTTCTCC	*cebp-1*
YJ11161	ctctcttccgattcctctcg	*nipi-3*
YJ12693	tattgaggcgagagctaggc	*mak-2*
YJ12692	acCGGACTAGCTCCACCTTT	*mak-2*
YJ12694	CGAATGGGATTGTGTTTCTG	*mak-2*
YJ12695	GCATCCGGCTTGTACAGTct	*sek-1*
YJ12697	CCGACTCCACGAGAAGGATA	*pmk-1*
YJ12698	acGATCAGATCCAGGGAACA	*pmk-1*
YJ12699	CGGGAGATAGAAGGCATctg	*tir-1*
YJ12700	CGGGGTTGATTCAGACACTT	*hda-4*
YJ12701	CGCTTGATCACCAAGGATCT	*natc-2*
YJ12702	TGAAACCGTCGAACATTCTG	*sek-1*
YJ12703	CTCATGCACATCTTCCAATGA	*nsy-1*
YJ12704	CGGCGTGTCTACAACTTTCA	*nsy-1*
YJ12705	tcgctgcgagatattttgtg	*natc-2*
YJ12706	acgattctcgtttcccacac	*natc-2*
YJ12707	tcatggccttattggaggag	*hda-4*
YJ12708	caaacagcgcgagtaacaag	*nipi-3*
YJ12709	acttcatatccgccaagtgc	*hda-4*
YJ12710	cgaggtgcatgttgtttacc	*natc-1*
YJ12711	TAATCGTCGGCGTAGAGTCC	*natc-1*
YJ12712	AGGAGCCGAAGAGAATTCTGAC	*natc-1*
YJ12713	GACCGAAAGTTCTCGCAAAG	*natc-1*
YJ12714	CACGACGTTTCAACTCTTCTCC	*natc-1*
YJ12715	CCTCCTCATCCGGAGTATCA	*natc-2*
YJ12716	gtctcgaagcttccacttgc	*natc-2*
YJ12717	cgatttgcttaagtctcggttc	*sek-1*
YJ12718	tgacatcgaaaatggcactg	*sek-1*
YJ12719	tttcagGAAATCGGTGCAAT	*tir-1*
YJ12720	tctggaaaacagccaacaga	*nsy-1*
YJ12721	catcaacggttgtacttcctca	*nsy-1*
YJ12722	ggcgggaacctacttcactt	*natc-3*
YJ12723	cacggggacttcgtttacca	*natc-3*
YJ9980	acgcaggtcactcgtttc	*sek-1*
AC2396	gcagtgactgaatcgaaacga	*nsy-1*
AC2397	acacggaactcgtagtgactg	*nsy-1*
AC2398	atccacgtagccaactgacaa	*nsy-1*
AC2408	ctgtacggatacggaagaaga	*pmk-1*
YJ12609	tcggattcaggagcgaggtcag	*mak-2*
YJ10840	gggcattgggtaaatgagg	*tir-1*
YJ10841	AGCCTTCTGCATCCACAACT	*tir-1*
YJ10842	gccagctgtcaataccgttt	*tir-1*

Supplementary Table S6. CRISPR crRNA sequences

crRNA Target	Sequence
hda-4 5'	3'-GGATGAGTAGGAATAAGGCA-5'
hda-4 3'	5'-GGACACCCGAACGCACTTGG-3'
natc-2 5'	3'-GGCAAGTTCATCGTTGCGTG-5'
natc-2 3'	5'-GGATGATACTCCGGATGAGG-3'
natc-3 5'	3'-GGAAGCCCATCTGAAAGTGCA-5'
natc-3 3'	5'-TGAAAGGAGTCCGATTTCTA-3'

Supplementary References

Kim, K. W. *et al.* **Coordinated inhibition of C/EBP by Tribbles in multiple tissues is essential for *Caenorhabditis elegans* development**. *BMC Biol* 2016, **14**, 104.

Sarov M, Schneider S, Pozniakovski A, Roguev A, Ernst S, Zhang Y, Hyman AA, Stewart AF. **A recombineering pipeline for functional genomics applied to Caenorhabditis elegans**. *Nat Methods* 2006, **3**(10):839-844.

van der Linden AM, Nolan KM, Sengupta P. **KIN-29 SIK regulates chemoreceptor gene expression via an MEF2 transcription factor and a class II HDAC**. *EMBO J* 2007, **26**(2):358-370.

Dickinson DJ, Pani AM, Heppert JK, Higgins CD, Goldstein B. **Streamlined Genome Engineering with a Self-Excising Drug Selection Cassette**. *Genetics* 2015, **200**(4):1035-1049.
